# Grapevine bZIP transcription factor bZIP45 regulates *VvANN1* and confers drought tolerance in *Arabidopsis*

**DOI:** 10.3389/fpls.2023.1128002

**Published:** 2023-02-09

**Authors:** Shuaike Niu, Xiangyang Gu, Qian Zhang, Xuemin Tian, Zhan Chen, Jingru Liu, Xiaoju Wei, Chengxiang Yan, Ziwen Liu, Xiaoji Wang, Zhengge Zhu

**Affiliations:** ^1^ Ministry of Education Key Laboratory of Molecular and Cellular Biology, Hebei Research Center of the Basic Discipline of Cell Biology, Hebei Collaboration Innovation Center for Cell Signaling and Environmental Adaptation, Hebei Key Laboratory of Molecular and Cellular Biology, College of Life Sciences, Hebei Normal University, Shijiazhuang, China; ^2^ Grape Breeding, Shijiazhuang Institute of Pomology, Hebei Academy of Agriculture and Forestry Sciences, Shijiazhuang, China

**Keywords:** grapevine, annexin, bZIP transcription factor, drought stress, ROS

## Abstract

Drought is a severe environmental condition that restricts the vegetative growth and reduces the yield of grapevine (*Vitis vinifera L.*). However, the mechanisms underlying grapevine response and adaptation to drought stress remain unclear. In the present study, we characterized an ANNEXIN gene, *VvANN1*, which plays a positive role in the drought stress response. The results indicated that *VvANN1* was significantly induced by osmotic stress. Expression of *VvANN1* in *Arabidopsis thaliana* enhanced osmotic and drought tolerance through modulating the level of MDA, H_2_O_2_, and O_2_^·-^ at the seedling stage, implying that *VvANN1* might be involved in the process of ROS homeostasis under drought or osmotic stress conditions. Moreover, we used yeast one-hybridization and chromatin immunoprecipitation assays to show that VvbZIP45 could regulate *VvANN*1 expression by directly binding to the promoter region of *VvANN*1 in response to drought stress. We also generated transgenic *Arabidopsis* that constitutively expressed the *VvbZIP45* gene (35S::*VvbZIP45*) and further produced *VvANN1Pro*::GUS/35S::*VvbZIP45 Arabidopsis* plants *via* crossing. The genetic analysis results subsequently indicated that VvbZIP45 could enhance GUS expression *in vivo* under drought stress. Our findings suggest that VvbZIP45 may modulate *VvANN1* expression in response to drought stress and reduce the impact of drought on fruit quality and yield.

## Introduction

Drought stress is a major abiotic stress that reduces the yield and quality of plants during plant growth and development (Zhu, 2016). The expression of drought-related genes increased under drought stress, promoting a series of physiological, biochemical, and molecular reactions (Kooyers, 2015). Grapevine (*Vitis vinifera L.*) is an important fruit crop worldwide. Its fruit is used to produce wine, grape juice, and other foods (Ali et al., 2010; Liu et al., 2021a). However, the development of the grape industry and global climate change have led to grapevine cultivation lands being constantly subjected to drought, high temperature, cold, salt, and other abiotic stresses. Drought stress is a major constraint on grapevine productivity and quality (Hou et al., 2020; Li et al., 2021). Grapevines have evolved various mechanisms at the morphological, physiological, biochemical, and molecular levels in response to drought stress (Lovisolo et al., 2010; Romero et al., 2012). Moreover, several drought-related genes have been identified in grapevine. The expression of *VaNAC17* was induced by drought stress and substantially enhanced drought tolerance in transgenic *Arabidopsis thaliana* (Su et al., 2020). Heterologous expressing of *VaNAC26* in *Arabidopsis* improved drought tolerance by up-regulating drought stress-related genes and jasmonic acid (JA) signaling genes (Fang et al., 2016). Ectopic expression of *VaCIPK02* in *Arabidopsis* enhanced drought resistance by regulating abscisic acid (ABA) signaling and production reactive oxygen species (ROS) (Xu et al., 2020). Identification and characterization of candidate genes associated with drought stress can be utilized to enhance drought stress response in grape varieties, thus improving grapevine yield.

Drought stress often leads to increased production of ROS, including hydrogen peroxide (H_2_O_2_), superoxide ions (O_2_^·-^), and hydroxyl radicals (OH^•^) (Cruz De Carvalho, 2008). High ROS concentrations could damage cellular compounds such as proteins, membranes, and cellular RNA and DNA (Apel and Hirt, 2004; Miller et al., 2010). Therefore, plants have evolved an enzymatic antioxidant defense system to maintain cellular ROS homeostasis under various stress conditions. The main plant ROS-scavenging enzymes include superoxide dismutase (SOD), ascorbate peroxidase (APX), catalase (CAT), glutathione peroxidase (GPX), and peroxiredoxin (PrxR) (Mittler et al., 2004). Previous studies have indicated that increased expression of ROS scavenging-related genes could increase tolerance to drought stress. For example, overexpression of *OsLG3* increased drought stress tolerance in rice by inducing the expression of ROS scavenging genes (Xiong et al., 2018). Overexpression of *SlbHLH22* improved tomato plant drought stress tolerance by improving ROS scavenging system (Waseem et al., 2019). *VvWRKY13* negatively modulates plant drought tolerance through regulating the activities of CAT and SOD (Hou et al., 2020). Therefore, by studying ROS levels and antioxidant enzyme activities in plants under drought stress, we can deeply reveal the function of drought stress tolerance related genes.

Annexins are conserved Ca^2+^-dependent phospholipid-binding proteins that exist in plants, animals, and fungi (Rescher and Gerke, 2004; Mortimer et al., 2008). Previous studies have shown that, besides having peroxidase and ATPase/GTPase activities and responding to various abiotic stresses, annexins could mediate calcium transport in plants (Laohavisit and Davies, 2011; Clark et al., 2012; Demidchik et al., 2018; Saad et al., 2020). *OsANN1* and *OsANN10* conferred tolerance to abiotic stress in rice by modulating ROS levels (Qiao et al., 2015; Gao et al., 2020). *AtANN1* regulated [Ca^2+^]_cyt_ elevation in response to salinity stress and also participated in drought stress tolerance by regulating ROS production (Laohavisit et al., 2013). *OsANN4* activated Ca^2+^ influx in response to ABA (Zhang et al., 2021). *ZmANN33* and *ZmANN35* were up-regulated and participated in plasma membrane (PM) recovery during seed germination. In addition, inhibiting the expression of *ZmANN33* and *ZmANN35* increased membrane damage under chilling stress (He et al., 2019). However, a few reports exist on the roles of ANNEXINs in grapevine under abiotic stresses (Jami et al., 2012; Briz-Cid et al., 2016). Investigation the role of grapevine ANNEXINs involvment in stress response will enrich the function of annexin in different species.

The dynamic balance between plant growth regulation and stress adaptive response is implicated by many regulatory proteins, among which transcription factors (TFs) are key components that modulate stress adaptation pathways in plant stress responses. The basic leucine zipper (bZIP) TFs belong to one of the largest transcription factor families and are characterized by a basic DNA-binding region with a specific motif (N-X7-R/K) at the N-terminus and leucine zipper region at the C-terminus. They play pivotal and diverse roles in plants under high-temperature, salt, and drought stress conditions (Corrêa et al., 2008; Nijhawan et al., 2008). *OsbZIP62V* significantly enhanced tolerance to drought and oxidative stress, and the *osbzip62* mutants displayed reduced drought stress tolerance (Yang et al., 2019). *Zea mays bZIP60* mediated the unfolded protein response during heat stress (Li et al., 2020). The wilting degree was noticeably lower in *TabZIP15* overexpressing plants than in KN199 plants under salt treatment (Bi et al., 2021). HvbZIP21 play a key role in drought stress tolerance through modulating ROS scavenging (Pan et al., 2022). The bZIP TFs can bind to the core sequence (-ACGT-) in the promoter of downstream genes (e.g., the G-box, C-box, and A-box), thereby participating in the transcriptional regulation of plant responses to stress (Nijhawan et al., 2008). The maize bZIP TF bZIP68 acts as a negative regulator of cold tolerance and directly binds to the A-box/G-box in the *DREB1.7* promoter, inhibiting the expression of the *DREB1* gene (Li et al., 2022). To date, 55 bZIP genes have been identified in grapevines, of which 32 *VvbZIP* genes are widely involved in responding to drought stress (Liu et al., 2014). *VvbZIP45*, also named *VvGRIP55* or *VvABF2*, could bind to the ABA-responsive element and play a positive role in response to drought stress (Nicolas et al., 2014; Liu et al., 2019).

In the present study, we aimed to isolate and characterize a grapevine putative annexin gene, *VvANN1* (Vv18g03470). Our results showed that heterologous expression of grapevine *VvANN1* improved drought stress tolerance in *Arabidopsis via* reducing malondialdehyde (MDA) and increasing the activities of SOD, POD, and CAT in leaves under drought conditions. In addition, we found that drought-responsive TF VvbZIP45 regulated the expression of *VvANN1*, thus improving drought resistance in grapevine. We reveal a working mechanism of VvbZIP45-mediated *VvANN1* in response to drought stress that may reduce the impact of drought on fruit quality and yield.

## Materials and methods

### Plant materials and growth conditions

Grapevine (*V. vinifera* L. cv. ‘Summer Black’ and ‘Venus’) was used in this study. Plantlets were grown on solid Murashige and Skoog (MS) medium under a 16-h light/8-h dark cycle and 70% relative humidity at 25°C in the greenhouse.

All transgenic lines of *VvANN1* were developed in the *A. thaliana* Columbia (Col-0) background. Col-0 plants were used as the wild type in the present study. *Arabidopsis thaliana* seedings were germinated on MS for 6 days and transferred to soil pots (7cm×7 cm). The seedlings were grown in a light incubator (22°C, 16-h day/8-h night cycle and 70% relative humidity).

### Phenotypic analysis

Five-week-old plantlets were transferred to liquid MS medium for 2 days and then planted in a fresh liquid MS medium with 10% (w/v) PEG6000 (NO. A610432, Sangon Biotech, Shanghai, China) to evaluate osmotic stress tolerance. Stem apex samples were collected for quantitative real-time polymerase chain reaction (RT-qPCR) analysis.

Six-day-old *VvANN1* transgenic and Col-0 seedlings were germinated on MS medium, transferred to soil pots (7 cm×7 cm) for 9 days with regular water. These were dried for 7 days and then allowed a 3-day recovery. The survival rates were recorded and the seedings were photographed.

### Vector construction

The total cDNA of *VvANN1* and a 1,538-bp fragment of the *VvANN1* promoter were cloned from five-week-old grapevine ‘Summer Black’ plantlets. The *VvANN1* coding region sequence (cDNA) was digested and ligated with pCAMBIA1301-HA, modified pMDC83, and pET32a vectors to obtain Ubi::*VvANN1*-HA, 35S::*VvANN1*-GFP, and *VvANN1*-His constructs, respectively.

The promoter region (1,538 bp) upstream of the *VvANN1* start codon was amplified and cloned into the vector to produce transgenic *VvANN1Pro*::GUS lines. Genomic fragments (285 bp) upstream of *VvANN1* were amplified and cloned into pAbAi and pGreenII 0800-LUC vectors to generate pAbAi-*VvANN1Pro* and pGreenII 0800-LUC-*VvANN1Pro*, respectively.

*VvbZIP45* cDNA was amplified and cloned into pCAMBIA1301-HA, pGreenII 62-SK, pGADT7 and pMDC83 vectors to generate Ubi::*VvbZIP45*-HA, pGreenII 62-SK-*VvbZIP45*, AD-*VvbZIP45* and 35S::*VvbZIP45*-GFP, respectively.

Related constructs were introduced into *Agrobacterium tumefaciens* cells (GV3101) and grown at 28°C for 3 days before being transformed into Col-0. The primers used to produce the constructs are listed in **Supplemental Table S1**.

### RNA isolation and RT-qPCR analysis

Total RNA was extracted from grapevine stem apices and *Arabidopsis* leaves using TRIzol reagent (NO. B511311, Sangon Biotech, Shanghai, China). RNase-free DNase I (EN0521, Thermo Fisher Scientific, Waltham, MA, USA) was used to remove genomic DNA. RT-qPCR was performed using the ChamQ Universal SYBR^®^ qPCR Master Mix Kit (Q711-02, Vazyme, Nanjing, China) using the QuantStudio Q5 thermal cycler (Thermo Fisher Scientific, Waltham, MA, USA). The experiment was performed using three biological replicates. Relative quantitative results were calculated by normalization to *AtACTIN2* and *VvACTIN7*, the internal controls in *Arabidopsis* and grapevine, respectively. All primer sequences are listed in **Supplemental Table S1**.

### Histochemical β-glucuronidase (GUS) assay

GUS activity was detected *via* histochemical staining of tissues as previously described (Zhang et al., 2018a) but with slight modifications. All transgenic *Arabidopsis* tissues were incubated in GUS staining solution at 37°C with the corresponding time under dark conditions. All stained samples were washed with 70% ethanol to remove the residual dye and chlorophyll. Images were captured using a DVM6a 3D microscope (Leica, Wetzlar, Germany).

### Subcellular localization of VvANN1

Transient expression assays were performed in grape protoplasts to determine the subcellular localization of VvANN1. Grape protoplasts were prepared, and transient expression assays were performed as described previously (Lee and Wetzstein, 1988; Wang et al., 2015). The recombinant plasmid 35S::*VvANN1*-GFP was introduced into grape protoplasts, followed by incubation at 28°C for 12 h. Fluorescence signals were observed using a Zeiss LSM710 laser scanning confocal microscope (Zeiss, Jena, Germany).

### Recombinant VvANN1-His protein purification and Ca^2+^-binding activity

The Ca^2+^-binding assay was conducted by detecting fluorescence measurements of VvANN1 according to a method described previously (Qiao et al., 2015). The recombinant plasmid *VvANN1*-His was introduced into *Escherichia coli* (*E. coli*) strain BL21. Total VvANN1-His protein was induced by isopropyl β-D-thiogalactoside (IPTG; NO. A100487, Sangon Biotech, Shanghai, China) at 28°C for 12 h. *E. coli* cells were lysed *via* ultra-sonication, and the obtained samples were ultra-centrifuged at 12,000 ×*g* at 4°C for 15 min. The supernatant was collected and purified *via* affinity chromatography on Ni-agarose columns (Cat. No. 30210, Qiagen, Duesseldorf Germany). The assay media contained 2 μM VvANN1-His protein and 0 mM or 2 mM Ca^2+^. Fluorescence spectroscopy was carried out using a fluorescence spectrophotometer (F-4600; Hitachi, Tokyo, Japan).

### Yeast one-hybrid assay

The pAbAi-*VvANN1Pro* (containing 285 bp partial promoter sequences with the ABRE element) vector was transformed into the yeast strain Y1HGold as bait. Positive yeast strains were diluted and spread onto selection medium (SD; Code No:630411, Clontech, Mountain View, CA, USA) lacking Ura containing various concentrations of Aureobasidin A (AbA; Code No.630466, Clontech, Mountain View, CA, USA) to screen for an appropriate concentration to eliminate self‐activation. AD*-VvbZIP45* was transformed into Y1HGold with pAbAi-*VvANN1Pro*, and the pGADT7 plasmid was used as a negative control. Different experimental groups were cultured on SD/-Leu medium with or without 80 ng/mL AbA at 30°C for 3 days.

### Luciferase reporter assays

A dual-luciferase reporter assay was conducted to test the transcriptional repression activity of VvbZIP45 in tobacco (*Nicotiana tabacum*) leaves. The pGreenII 62-SK and pGreenII 62-SK-*VvbZIP45* were used as effector plasmids, and pGreenII 0800-LUC-*VvANN1Pro* was used as the reporter plasmid. The plasmids were mixed and expressed in tobacco leaves *via A. tumefaciens* GV3101 strain injection. After 2 days, total protein was extracted from infiltrated tobacco leaves, and the LUC/REN activity ratio was measured using the dual luciferase reporter assay system (E1960, Promega, Madison, WI, USA).

### Chromatin Immunoprecipitation-qPCR assays

Approximately 1 g of plant tissue was harvested from two-week-old transgenic hybrid progeny seedlings that were germinated on MS plates in a light incubator. Samples were prepared according to previous reports (Yamaguchi et al., 2014; Zhao et al., 2022). ChIP experiments were performed using Abcam ChIP Kit - Plants (ab117137, Abcam, Cambridge, MA, USA) according to the manufacturer’s instructions. Chromatin was immunoprecipitated using anti-HA (ab9110, Abcam, Cambridge, MA, USA). Following immunoprecipitation, samples were analyzed by RT-qPCR. The specific primers used are listed in **Supplemental Table S1**.

### Detection of H_2_O_2_ and O_2_^·-^ *in situ*

Leaves were collected from *VvANN1* transgenic *Arabidopsis* plants and Col-0 plants grown under normal conditions for 15 days, then grown with or without water for 5 days. H_2_O_2_ and O_2_^·-^ levels were examined *via* histochemical staining with 3, 3′‐diaminobenzidine (DAB; CAS No: 91-95-2, Sigma-Aldrich, St. Louis, MO, USA) or nitro blue tetrazolium (NBT; CAS No: 298-83-9, Sigma-Aldrich, St. Louis, MO, USA), as previously described (Zhang et al., 2021). All measurements were determined using three independent biological replicates.

### Measurement of antioxidant enzyme activity and MDA contents

Leaves were collected from *VvANN1* transgenic *Arabidopsis* plants and Col-0 plants cultivated under normal conditions for 15 days (control), then without water for 5 days (drought-exposed).

Samples (each weighing 0.2 g) were homogenized in 1 mL of sodium phosphate buffer (50 mM phosphate, 1 mM EDTA-Na_2_, 1% (w/v) polyvinyl pyrrolidone; pH 7.4). Centrifugation was performed at 10,000 ×*g* at 4°C for 20 min, and the supernatant was used to detect antioxidant enzyme activity. The activities of SOD, POD, and CAT were determined according to methods described previously (Zhang et al., 2017).

Samples (each weighing 0.2 g) were homogenized in 2 mL of 10% thiobarbituric acid (TBA). Following centrifugation at 12,000 ×*g* at 4°C for 15 min, the MDA contents were detected according to the method described by Zhou et al. (2018).

## Results

### Characterization and expression of *VvANN1*

**Figure 1:**
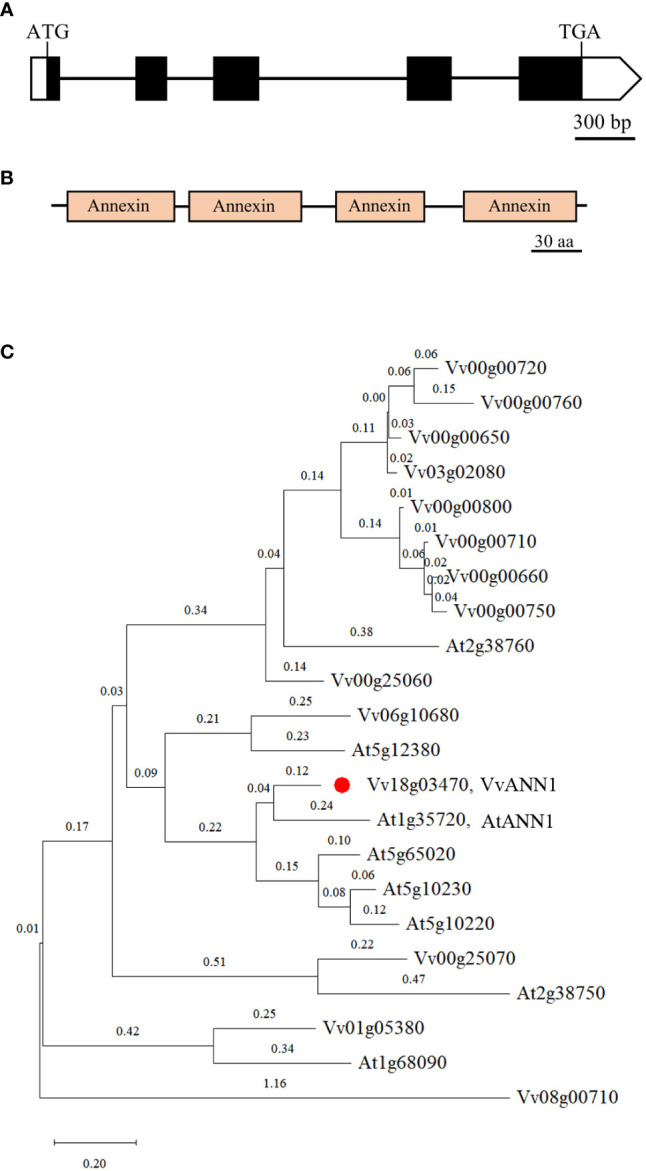
Characterization of VvANN1. **(A, B)** Schematic diagram showing the gene structure and protein domain of VvANN1. Exons are indicated by black boxes, introns are indicated by black lines between black boxes, non-translational regions are indicated by white boxes, and annexin domains are indicated by orange boxes. **(C)** Phylogenic tree of VvANNs and the Arabidopsis ortholog AtANNs. The phylogenetic trees were constructed with Mega X using the Neighbor-Joining method. Eight ANNEXINs family members from *A thaliana* including At1g35720 (AtANN1), At5g65020, At2g38760, At2g38750, At1g68090, At5g10220, At5g10230, At5g12380; fourteen ANNEXINs family members from *V. vinifera* including: Vv18g03470 (LOC100266093, VvANN1), Vv00g00650 (LOC100260243), Vv00g00660 (LOC100258538), Vv00g00710 (LOC100244780), Vv00g00720 (LOC100853064), Vv00g00750 (LOC100244780), Vv00g00760(LOC100256827), Vv00g00800 (LOC100260252), Vv00g25060 (LOC100256917), Vv00g25070 (LOC100253408), Vv01g05380 (LOC100250931), Vv03g02080 (LOC100243369), Vv06g10680(LOC100263694), Vv08g00710(LOC100240986), VvANN1 is marked with a red dot. The sequences of AtANNs are derived from NCBI, and the sequences of VvANNs are derived from Jami et al (2012).

There are 14 predicted annexin genes in grapevine (Jami et al., 2012), however, the function of ANNEXIN family in grapevine remains unidentified. We isolated a putative grapevine annexin gene from *Vitis vinifera via* RT-PCR and named it *VvANN1.* Based on the results of bioinformatics analyses, the genomic sequence of *VvANN1* is composed of five exons and four introns and produces a 930-bp coding sequence transcript encoding a protein of 309 amino acids with a molecular weight of 35 kDa (**Figure 1A**). This protein contains four annexin domain architectures (**Figure 1B**) with a type-II Ca^2+^ binding site in the first annexin conserved repeat domain.

A phylogenetic tree generated using the ANNEXINs of *A. thaliana* and *V. vinifera* suggests that *VvANN1* has the maximum homology with *AtANN1* of *A. thaliana*, so the gene was designated as *VvANN1*. This finding implies that VvANN1 proteins may regulate stress response processes similar to its homologous *AtANN1* (**Figure 1C**).

**Figure 2:**
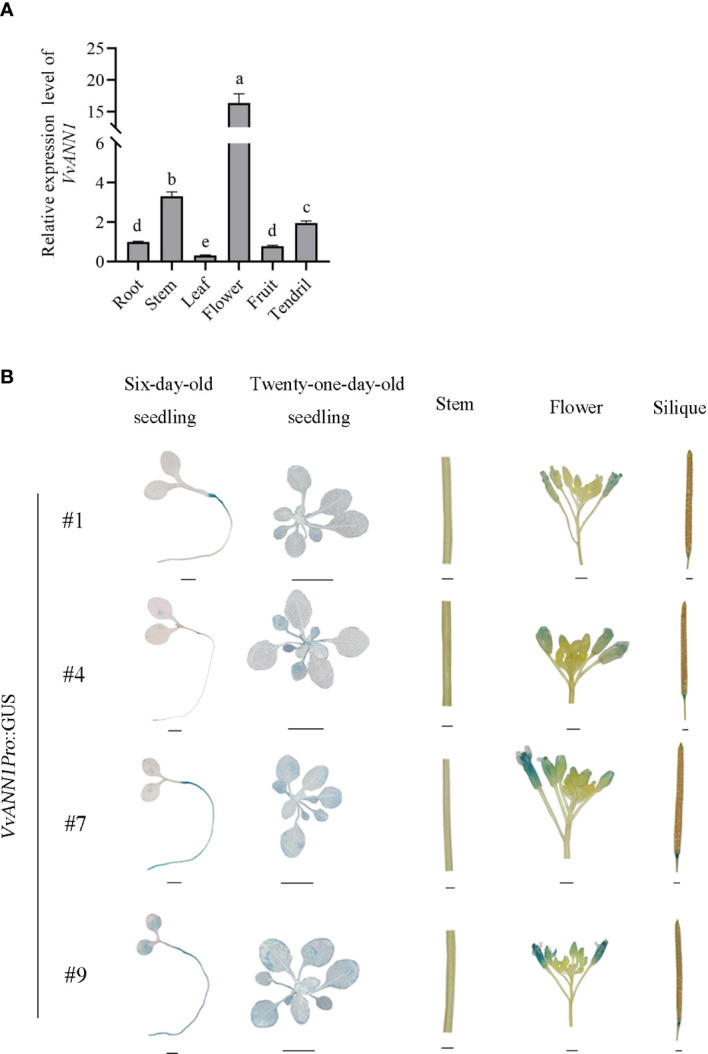
Tissue expression pattern analysis of *VvANN1*. **(A)** Expression analysis of *VvANN1* in different tissues of *Vitis* spp. cv ‘Venus’ *via* RT-qPCR. *VvACTIN7* was used as an internal control and compared to expression in root. Data are presented as mean ± SD (n = 3). Statistical significance was determined *via* one-way ANOVA; P < 0.05. **(B)** GUS histochemical staining of different tissues of *VvANN1Pro*::GUS transgenic *Arabidopsis* lines. Scale bars are 1 mm for six-day-old seedling, flower, silique, and stem images and 1 cm for 21-day-old seedling images.

Transcript levels in different organs of the grapevine ‘Venus’ were performed *via* RT-qPCR to analyze the tissue-specific expression of *VvANN1*. The expression of *VvANN1* was higher in the flower, stem, and tendril than in the root, fruit, and leaf (**Figure 2A**). Furthermore, we also generated *Arabidopsis* transgenic lines (#1, #4, #7 and #9) containing the *VvANN1Pro*::GUS, and detected GUS activities in seedlings, flowers, which was consistent with the findings shown by RT-qPCR (**Figure 2B**). Moreover, important discrepancies were noted when comparing the GUS staining observed in different *VvANN1Pro*::GUS transgenic lines, such as #1 and #9. This result suggested the cassette *VvANN1*::GUS might be inserted various sites in different *Arabidopsis* transgenic lines and resulted in different GUS expression level based on the position effect, for example, there might be diverse enhancers near to the insertion sites.

We transformed the 35S::*VvANN1*-GFP vector transiently into grapevine protoplasts to investigate the subcellular localization of VvANN1. VvANN1-GFP signal could be detected in cytoplasm, whereas GFP signals were ubiquitously distributed in the grapevine protoplast (**Supplemental Figure S1**).

Furthermore, we carried out a VvANN1-His recombinant protein fluorescence experiment to verify the Ca^2+^ binding ability of VvANN1. First, we transformed the *VvANN1*-His plasmid into *E. coli* strain BL21. The VvANN1-His recombinant protein was successfully expressed in BL21 by adding IPTG, purified *via* Ni-NTA affinity chromatography, and further detected *via* SDS-PAGE (**Supplemental Figure S2**). Next, a UV spectrophotometer was used to measure the fluorescence intensity of the VvANN1-His recombinant protein when incubated with or without Ca^2+^. The highest fluorescence intensity was approximately 300 A.U. at 390 nm. By contrast, fluorescence intensity was reduced to 150 A.U. at 390 nm in the presence of Ca^2+^ (**Supplemental Figure S3**), indicating that VvANN1 might have Ca^2+^-binding capacity.

### *VvANN1* is responsive to osmotic stress and drought stress

*AtANN1* is up-regulated in a Ca^2+^-dependent manner to regulate drought stress responses synergistically. Here, we investigated whether *VvANN1* was responsive to drought stress. We performed RT-qPCR to detect the *VvANN1* expression pattern in plantlets (*Vitis* spp. cv ‘Summer Black’ and ‘Venus’) treated with or without 10% PEG6000. The results showed that the expression of *VvANN1* in ‘Summer Black’ and ‘Venus’ was significantly induced by PEG treatment up to 11-fold and 4-fold, respectively, at 24 h (**Supplemental Figure S4A**).

GUS staining was performed to determine further the expression of *VvANN1* in six-day-old transgenic *VvANN1Pro*::GUS plants under PEG treatment. Histochemical staining revealed that, *VvANN1Pro*::GUS signals were mainly expressed in the vascular tissues of roots under normal conditions. This expression pattern of GUS activity was increased following treatment with 10% PEG6000 for 12 h (**Supplemental Figure S4B**), suggesting that *VvANN1* expression may be induced by PEG treatment.

**Figure 3:**
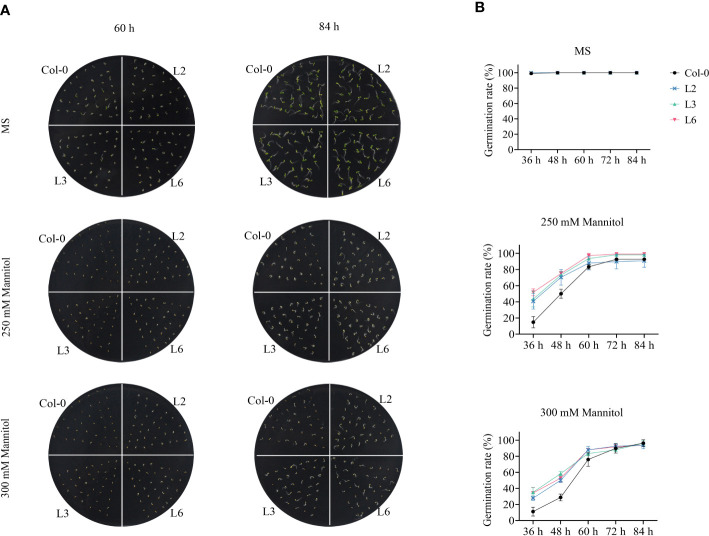
*VvANN1* positively regulates osmotic stress at germination in *Arabidopsis*. **(A)** Seedings of Col-0 and *VvANN1* transgenic *Arabidopsis* were stratified at 4°C for 2 days and plated on MS medium supplemented with 0 mM, 250 mM or 300 mM mannitol. Photographs were captured at 60 h and 84 h after germination. **(B)** Germination rates of seedings were determined with respect to radicle emergence when supplemented with 0 mM, 250 mM or 300 mM mannitol at the 84-h time point.

To further determine the role of *VvANN1* in modulating plant osmotic stress, we further generated 3 independent transgenic *Arabidopsis* lines driven by the CaMV35S promoter (L2, L3 and L4) and 3 independent transgenic *Arabidopsis* lines driven by the Ubi promoter (L5, L6 and L7). The transcript level of *VvANN1* was analyzed with RT-qPCR and three homozygous transgenic *Arabidopsis* lines (L2, L3 and L6) were used in subsequent experiments (**Supplemental Figure S5**). *VvANN1* transgenic and Col-0 seedlings were cultivated on MS medium with 0 mM, 250 mM or 300 mM mannitol. The germination rates showed no apparent difference between *VvANN1* transgenic and Col-0 seedlings in MS medium after 84 h. Conversely, all *VvANN1* transgenic seedlings showed higher germinating rates than Col-0 in the presence of mannitol (**Figures 3A, B**), suggesting that, during the germination stage, *VvANN1* transgenic seedlings were less sensitive to osmotic stress than Col-0 seedlings. Based on these results, we concluded that *VvANN1* might positively regulate osmotic stress in *Arabidopsis*.

**Figure 4:**
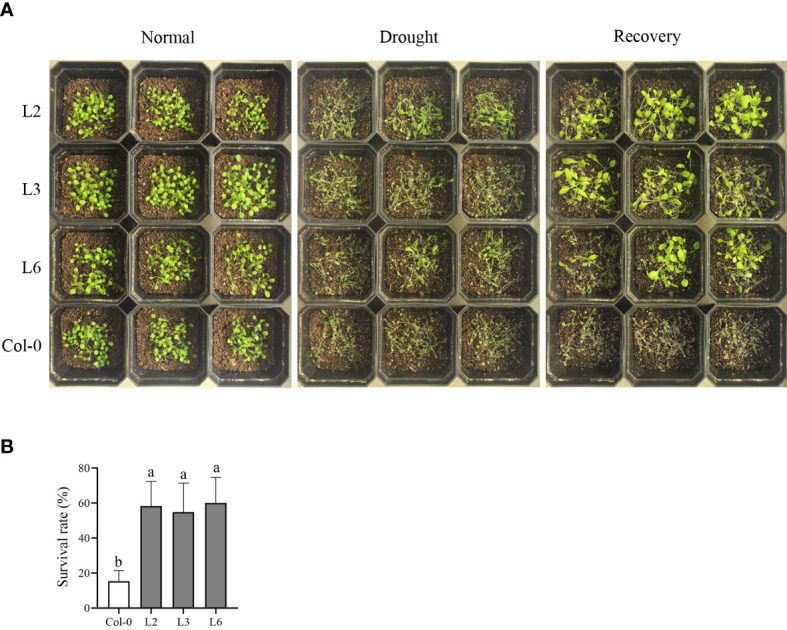
Heterologous expression of *VvANN1* increases resistance to drought stress in *Arabidopsis*. **(A)** Performance of *VvANN1* transgenic plants and Col-0 plants subjected to soil drought stress without watering for 7 days, followed by recovery for 3 days. **(B)** Survival rates of *VvANN1* transgenic plants and Col-0 plants after rewatering for 3 days. Values represent the means ± SD from three independent repeats (n = 48), and different letters indicate significant differences (one-way ANOVA, P < 0.05).

Fifteen-day-old transgenic *Arabidopsis* and Col-0 plants were subjected to a drought treatment to evaluate the function of *VvANN1* in drought tolerance. Once watering was stopped for 7 days, *VvANN1* transgenic plants exhibited less wilting than Col-0 plants (**Figure 4A**). After rewatering, *VvANN1* transgenic plants had higher survival rates (58%, 54% and 60% for L2, L3 and L6, respectively) than Col-0 plants (36%) (**Figure 4B**), indicating that *VvANN1* transgenic plants significantly improved drought tolerance in *Arabidopsis*.

### VvbZIP45 binds to the promoter of *VvANN1* and activates its expression

We analyzed the *VvANN1* promoter using the PlantCARE database (http://bioinformatics.psb.ugent.be/webtools/plantcare) to gain further insights into the regulatory mechanism of *VvANN1* and found several stress-responsive cis-elements (e.g., MYC, MYB and ABRE). Among these are two ABRE cis-elements in the 0- to 300-bp region of the *VvANN1* promoter. Studies have shown that VvbZIP45 can bind to the ABRE element in the promoter region of the target gene and enhance drought stress tolerance in *Arabidopsis* (Liu et al., 2014; Nicolas et al., 2014; Liu et al., 2019). Thus, we hypothesized that VvbZIP45 could bind to the promoter region of *VvANN1* and regulate its expression.

**Figure 5:**
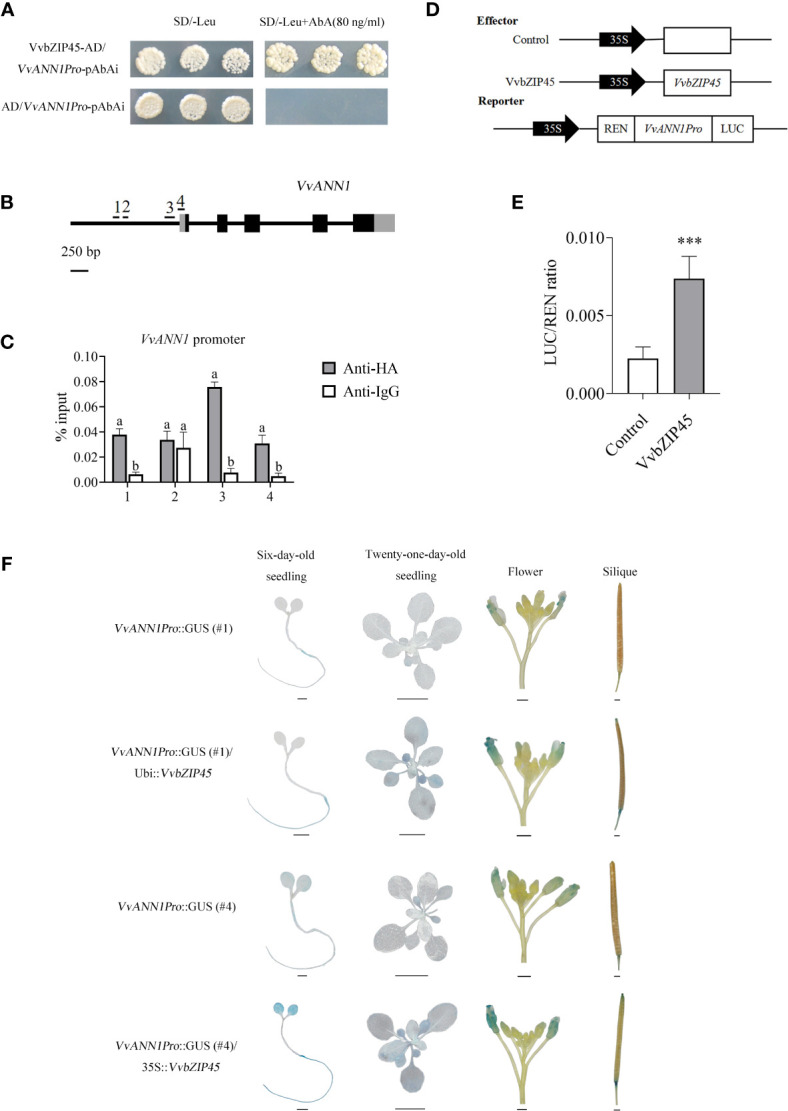
VvbZIP45 positively regulates the transcription of *VvANN1* by binding to its promoter. **(A)** Y1H assay showing the interaction between VvbZIP45 and the VvANN1 promoter. **(B, C)** ChIP-qPCR assays showing the binding of VvbZIP45 to the promoter of *VvANN1 in vivo*. ChIP-qPCR analysis using *VvANN1Pro*::GUS/UBI::VvbZIP45-HA and Col-0 plants with anti-HA and anti-IgG, respectively. Immunoprecipitated DNA samples were quantified by qPCR using primers specific to regions within the *VvANN1* promoter (1-4). Relative enrichment is represented as input (%). Values represent the means ± SD from three independent repeats, and different letters indicate significant differences (one-way ANOVA, P < 0.05). **(D)** Reporter and effector used in the dual-luciferase reporter assay. **(E)** Activation of the *VvANN1* promoter by VvbZIP45. The 35S promoter was used as a negative control (Student’s *t*-test, ***P < 0.001). **(F)** Tissue expression patterns of *VvANN1* in the presence of VvbZIP45 using VvbZIP45 as the effector and *VvANN1Pro*::GUS as the reporter. GUS expression was visualized in different tissues of *Arabidopsis* transformed with effector and reporter constructs. Scale bars are 1 mm for six-day-old seedling, flower, and silique images and 1cm for 21-day-old seedling images.

We performed a Y1H assay, where the *VvANN1* promoter sequence (from -1 to -285 bp) containing two ABRE motifs was first constructed into the pAbAi vector (pAbAi-*VvANN1Pro*). Next, this bait plasmid was transformed into yeast strain Y1HGold. Finally, the pGADT7-*VvbZIP45* and pGADT7 vectors were transformed into the yeast Y1HGold carrying the bait plasmid. The results showed that Y1HGold carrying pAbAi-*VvANN1Pro* and transformed with the pGADT7-*VvbZIP45* plasmid grew on SD/-Leu medium containing 80 ng/mL AbA, whereas yeast cells carrying the pGADT7 vector did not (**Figure 5A**). This result showed that VvbZIP45 could bind to the promoter region of *VvANN1*.

We performed a ChIP-qPCR assay to investigate whether VvbZIP45 could bind directly to the ABRE cis-element in the promoter region of *VvANN1*. Ubi::*VvbZIP45*-HA transgenic *Arabidopsis* plants were crossed with *VvANN1Pro*::GUS transgenic plant, and hybrid transgenic *A. thaliana* seedlings were verified *via* PCR. Four fragments spanning different regions of the *VvANN1* promoter with or without the ABRE motif were selected for qPCR analysis (**Figure 5B**). **Figure 5C** shows that 1, 3 and 4 fragments of the *VvANN1* promoter were markedly enriched in ChIP-qPCR assay with anti-HA compared with anti-IgG.

We used a luciferase reporting system to determine whether VvbZIP45 could activate *VvANN1* expression *in vivo* (**Figures 5D, E**). A pGreenII 0800 vector harboring a dual-luciferase reporter gene driven by the *VvANN1* promoter was co-transformed into tobacco leaves with pGreenII 62-SK or pGreenII 62-SK-*VvbZIP45*. The results showed that tobacco plants expressing pGreenII 62-SK-*VvbZIP45* exhibited significantly higher LUC/REN activity than control plants.

**Figure 6:**
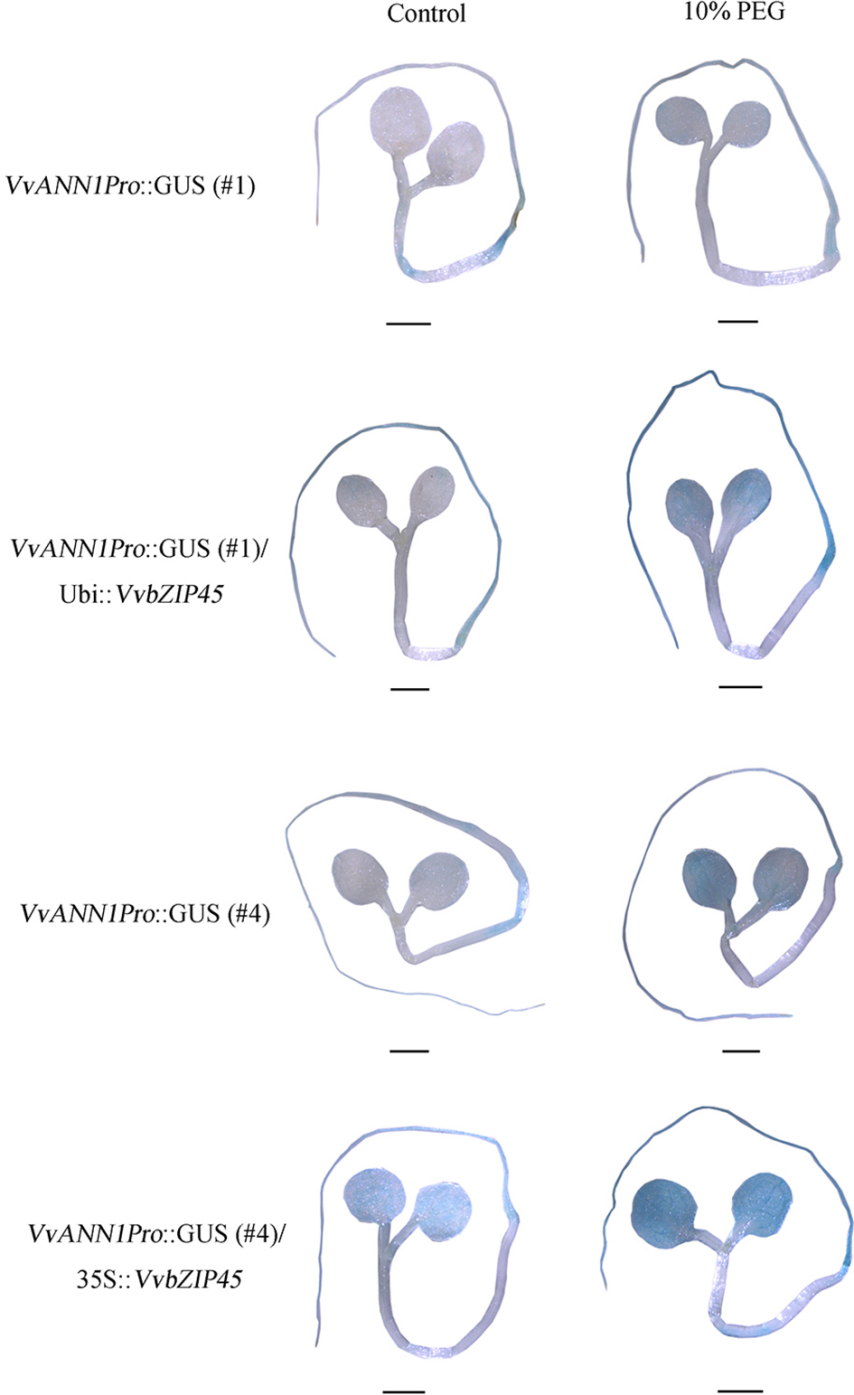
VvbZIP45 enhanced *VvANN1* expression under PEG treatment. GUS staining of six-day-old *VvANN1Pro*::GUS/35S::*VvbZIP45* and *VvANN1Pro*::GUS transgenic *Arabidopsis* seedlings under normal and PEG treatment. Scale bars=1 mm.

To further assure the regulation of VvbZIP45 in *VvANN1* expression *in vivo*, we also generated transgenic *Arabidopsis* that constitutively expressed the *VvbZIP45* gene (35S::*VvbZIP45*) and further produced *VvANN1Pro*::GUS/35S::*VvbZIP45 Arabidopsis* plants *via* crossing. The lower GUS expression level *VvANN1Pro*::GUS lines #1 and #4 (as shown in **Figure 2C**) were used as the male parents, 35S::*VvbZIP45 Arabidopsis* as female parent. The results of histochemical GUS assays showed that staining intensity was significantly higher in hybrid plants (*VvANN1Pro*::GUS/*VvbZIP45*) than in *VvANN1Pro*::GUS transgenic plants (**Figure 5F**). Interestingly, 10% PEG6000 treatment could be resulted in much more strong GUS staining in *VvANN1Pro*::GUS/35S::*VvbZIP45* hybrid *Arabidopsis* plants comparing to *VvANN1Pro*::GUS transgenic plants itself (**Figure 6**). These results indicated that VvbZIP45 could act as a transcription factor to promote *VvANN1* expression under normal or drought stress conditions.

Further examination of the expression level of VvbZIP45 in five-week-old grapevine plantlets under osmotic stress showed that *VvbZIP45* expression was rapidly activated by PEG treatment and reached the highest level at 12 h (**Supplemental Figure S6**). This result suggests that *VvbZIP45* could be up-regulated under drought stress.

### *VvANN1* improved the ROS scavenging ability of transgenic *Arabidopsis* under drought stress

**Figure 7:**
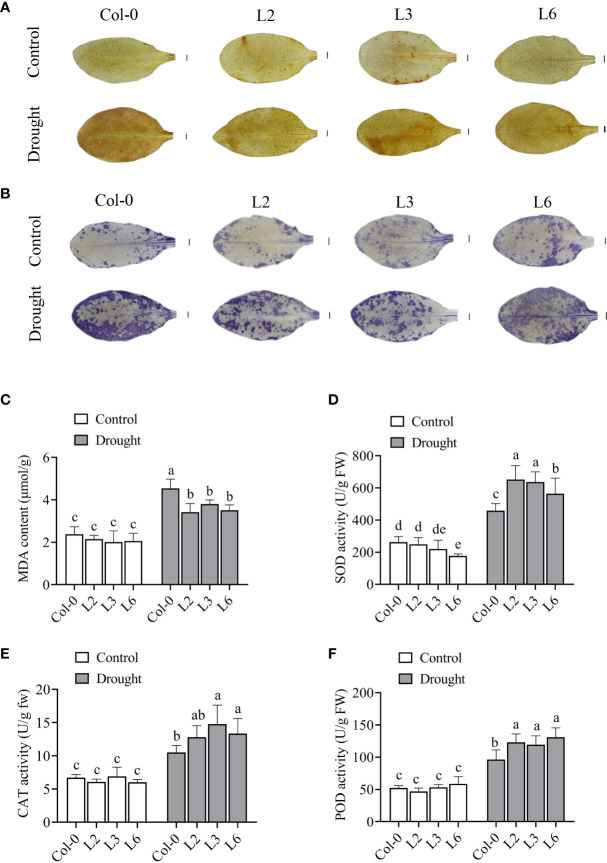
VvANN1 modulates ROS production, lipid peroxidation, and antioxidase activity under drought stress in *Arabidopsis*. **(A, B)** DAB and NBT staining of leaves in *VvANN1* transgenic plants and Col-0 plants under normal and drought stress conditions. Scale bars = 1 mm. **(C)** MDA content in *VvANN1* transgenic plants and Col-0 plants under normal and drought stress conditions. **(D–F)** Activities of SOD, CAT, and POD in *VvANN1* transgenic plants and Col-0 plants under normal and drought stress conditions. Values represent the means ± SD from three independent repeats, and different letters indicate significant differences (one-way ANOVA, P < 0.05).

In plants, ROS serve as signal molecules at low levels but can cause cell damage at extreme doses. Drought stress could lead to ROS accumulation. Here, H_2_O_2_ accumulation was detected *via* DAB staining. The results showed no difference in DAB staining of *VvANN1* transgenic plants and Col-0 plants under normal conditions. However, after 5 days of drought treatment, weaker staining was observed in *VvANN1* transgenic leaves than in Col-0 leaves (**Figure 7A**). The accumulation of O_2_^.-^ detected *via* NBT staining showed similar results under drought stress treatment (**Figure 7B**). These results indicated that less H_2_O_2_ and O_2_^.-^ were produced in *VvANN1* transgenic plants than in Col-0 plants under drought stress.

Previous studies have shown that ROS accumulation affects intracellular environment stability and severely damages the plant cell membrane (Zhang et al., 2018b). The level of MDA is an important indicators of cell membrane damage (Gao et al., 2020). In the present study, the MDA levels in *VvANN1* transgenic plants were similar to Col-0 plants under normal conditions. Following drought treatment for 5 days, three *VvANN1* transgenic plants had significantly lower MDA content (3.42, 3.80 and 3.51 μmol/g) than Col-0 (4.54 μmol/g) (**Figure 7C**). These results suggest that *VvANN1* plays a vital role in the reduced accumulation and damage of ROS in cells under drought stress.

The activities of ROS scavenging enzymes (SOD, POD and CAT) were examined to analyze the mechanism of *VvANN1* function in regulating the level of ROS (**Figures 7D–F**). The activities of SOD, POD, and CAT were not significantly different in both *VvANN1* transgenic plants and Col-0 plants under normal conditions. However, after drought stress treatment, the enzymatic activities of SOD, POD, and CAT were significantly higher in *VvANN1* transgenic plants than in Col-0 plants. These results suggested that the expression of *VvANN1* driven by CaMV35S or Ubi promoter in *Arabidopsis* could improve the activity of SOD, POD and CAT to eliminate excess ROS, subsequently assisting in the maintenance of plasma membrane integrity and improving drought stress tolerance.

## Discussion

Over the past few decades, the role of annexin in regulating abiotic and biotic stresses have been extensively studied in plants, especially in fruits and vegetables. For instance, *FaAnn5s* and *FaAnn8* were important in regulating plant hormone signaling during the growth and maturing of strawberry fruit (Chen et al., 2016). Furthermore, overexpression of *RsANN1a* in *Arabidopsis* enhanced heat tolerance, suggesting a key role in the heat stress response of radish (Shen et al., 2021). Additionally, *BnaANN* genes played important roles in JA signaling and multiple stress responses in *Brassica napu*s (He et al., 2020). However, the biological functions and regulatory mechanisms of ANNEXINs in grapevine remain unclear. This study will shed light on the roles of VvANN1 in drought stress in grapevine.

In the present study, we identified an annexin gene from *V. vinifera* and named it *VvANN1.* Sequence analysis and phylogenetic analysis showed that this protein contains four annexin domain architectures and has close homology with *ANN1* in *A. thaliana* (**Figures 1A–C**). Therefore, it is speculated that *VvANN1* and *AtANN1* might have similar functions. Furthermore, the different expression levels of *VvANN1* in grapevine tissues and ectopic expression *VvANN1* in *Arabidopsis* indicate that *VvANN1* may have distinct functions (**Figure 2**), so we firstly verified the ability of *VvANN1* to response to drought stress. As expected, the expression of *VvANN1* was induced by osmotic stress, and *VvANN1* transgenic plants showed higher germinating rates than Col-0 plants under osmotic stress (**Supplemental Figure S4** and **Figure 3**). In addition, overexpression of *VvANN1* enhanced drought tolerance in *A. thaliana* (**Figure 4**).

The bZIP TFs are widely distributed across several plant species and are involved in many responses to abiotic stresses, such as drought, salt, and low temperature (Wei et al., 2012; Liu et al., 2014; Li et al., 2015). For example, *OsbZIP23* positively regulates drought and high-salinity stress responses by modulating the expression of stress-related genes in rice (Xiang et al., 2008). ANAC096 interacts with AtABF2 and further regulates the expression of ABA-inducible genes, enhancing dehydration and osmotic stress tolerance in *Arabidopsis* (Xu et al., 2013). *VvbZIP45* transgenic *Arabidopsis* plants exhibited more tolerance to osmotic stress compared to WT (Liu et al., 2019). However, studies on the function of VvbZIP45 in regulating the expression of grapevine annexin genes have not been reported. Our results indicated that *VvbZIP45* expression in grapevine ‘Summer Black’ was significantly induced by PEG treatment (**Supplemental Figure S6**). Transient expression assays, Y1H assays, genetics investigations and ChIP-qPCR assays all showed that VvbZIP45 could bind to the promoter of *VvANN1* and activate its expression (**Figures 5A–E**).

As shown in **Figure 2C**, various *VvANN1Pro*::GUS lines showed differently level of GUS staining (strong or weak). This result implied that the cassette *VvANN1 Pro*::GUS might be inserted in various sites of chromosomes in *Arabidopsis*, and resulted in different expression level of GUS based on the position effect, for example, there might be diverse enhancers near to the insertion sites of T-DNA, and also reflected that there might be a certain range of regulation levels of *VvANN1* promoter by TFs in *Arabidopsis*. Therefore, the *VvANN1Pro*::GUS/35S::*VvbZIP45* hybrid lines showed more strong GUS staining intensity than that of the original *VvANN1Pro*::GUS transgenic lines (**Figure 5F**). In addition, the genetic analysis also demonstrated that VvbZIP45 could enhance the expression of *VvANN1* under PEG treatment (**Figure 6**). Taken together, these results indicated that VvbZIP45 could bind to the promoter of *VvANN1* and further active its expression under drought stress.

Drought stress often results in excessive ROS accumulation (Mahmood et al., 2019). A low ROS concentration serves as a signal in regulating plant growth and stress responses; however, excessive ROS accumulation can destroy cellular compounds. The antioxidant defense system is a crucial way to balance excess ROS in plants (You and Chan, 2015; Hussain et al., 2019). Plant annexins have been shown to exhibit the ability to respond to abiotic stresses by modulating ROS formation. For instance, overexpression of *OsANN1* in rice exposed to heat stress conditions enhanced the activities of SOD and CAT, decreased the content of H_2_O_2_, and improved the plant’s tolerance to heat (Qiao et al., 2015). Treatment with exogenous ABA showed significantly higher levels of O_2_^.–^ and H_2_O_2_ in the mesophyll cells of *OsANN4*-RNAi lines than in the WT (Zhang et al., 2021). Our results were broadly in line with those of previous studies; for example, the contents of O_2_^.–^ and H_2_O_2_ in *VvANN1* transgenic plants were lower than Col-0 plants under drought stress (**Figures 7A, B**). The activities of SOD, POD and CAT in *VvANN1* transgenic plants were significantly higher than in Col-0 (**Figures 7C–F**). Therefore, we hypothesize that *VvANN1* responds to drought stress, at least in part by modulating ROS accumulation. In this study, we have not identified a direct relationship between H_2_O_2_ content and the function of VvANN1. However, our results imply that VvANN1 may play crucial role on regulation the intracellular level of H_2_O_2_.

Ca^2+^ acts as a second messenger in plants and regulates the activation of a wide range of downstream processes in response to environmental and developmental stimuli (Xi et al., 2017; Tong et al., 2021). Ca^2+^ influx is primarily dependent on ion channels, such as the cyclic nucleotide-gated channel (CNGC) or glutamate receptor-like (GLR). Annexins were shown to function as Ca^2+^-permeable transporters based on their Ca^2+^-binding ability. *AtANN1* plays a positive regulatory role in response to cold stress by mediating cold-triggered Ca^2+^ influx; moreover, the [Ca^2+^]_cyt_ elevation was reduced in *atann1* mutants (Liu et al., 2021b). MYB30 regulates the oxidative and heat stress responses through *AtANN1* and *AtANN4* by mediating Ca^2+^ signals (Liao et al., 2017). *ZmANN33* and *ZmANN35* were involved in Ca^2+^ signaling transduction processes under chilling stress (He et al., 2019). Although we found that *VvANN1* was capable of Ca^2+^-binding activity (**Supplemental Figure S3**), the exact mechanism underlying how *VvANN1* regulates Ca^2+^ under drought stress remains to be further explored.

**Figure 8:**
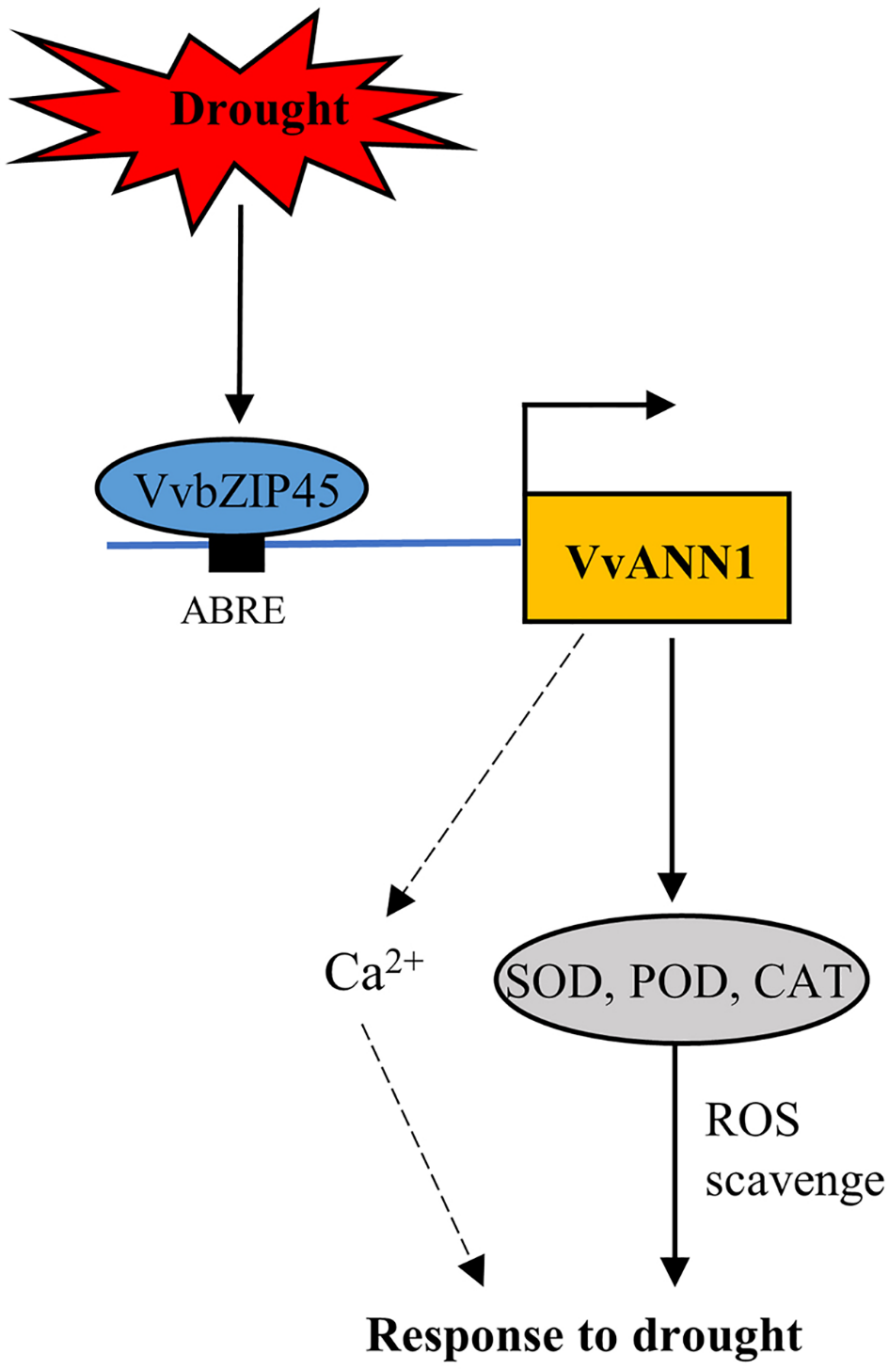
A hypothetical working model of VvbZIP45 modulates *VvANN1* response to drought stress. Drought stress induces the expression of *VvbZIP45* in grapevine, which then positively regulates the transcription of *VvANN1*. VvANN1 enhances the activity of SOD, POD, and CAT to scavenge excess ROS. *VvANN1* may also participate in Ca^2+^ signaling transduction in grapevine under drought stress.

We propose a hypothetical model depicting the role of *VvANN1* in response to drought stress based on our findings (**Figure 8**). The ABRE binding protein VvbZIP45 directly binds to the promoter region of *VvANN1* and activates its expression, thus further modulating ROS to alleviate the damage caused by drought stress. Our study provides insights into the roles of ANNEXINs in regulating drought responses in grapevine.

## Data availability statement

The raw data supporting the conclusions of this article will be made available by the authors, without undue reservation.

## Author contributions

ZZ and SN designed this study. SN performed most of the experiments. XG, QZ, XT, ZC, JL, XW, CY, ZL, and XW assisted in some experiments. ZZ, SN, and XG wrote the manuscript. All authors contributed to the article and approved the submitted version.

## Supplementary material

Supplementary Figure 1 Subcellular localization of VvANN1-GFP. Subcellular localization of VvANN1-GFP in grape protoplasts. Protoplasts transformed with 35S::GFP were used as control. Scale bars=20 μm.

Supplementary Figure 2 Affinity purification of VvANN1-His protein. Purification of VvANN1-His recombinant protein was performed with 0.2 mL elution buffer containing 500 mM imidazole (lane 1), lane 2 shows the molecular weight marker.

Supplementary Figure 3 The Ca^2+^-binding capacity of VvANN1-His. Fluorescence intensity of VvANN1-His without (black curve) or with (grey curve) the addition of 2 mM CaCl_2_.

Supplementary Figure 4 Expression patterns of *VvANN1* under PEG treatment. **(A)** RT-qPCR analysis of *VvANN1* expression in the five-week-old *Vitis* spp. cv ‘Summer Black’ and ‘Venus’ plantlets after 10% PEG6000 treatment. *VvACTIN7* was used as an internal control and compared to expression in 0 h. Values represent the means ± SD from three independent repeats, and different letters indicate significant differences (one-way ANOVA, P<0.05). **(B)** GUS staining of six-day-old *VvANN1Pro*::GUS transgenic *Arabidopsis* seedlings under normal and PEG treatment. Scale bars=1 mm.

Supplementary Figure 5 RT-qPCR analysis of *VvANN1* expression in different *VvANN1* transgenic *Arabidopsis* lines. *AtACTIN2* was used as an internal control. Values represent the means ± SD from three independent repeats.

Supplementary Figure 6
*VvbZIP4*5 transcript expression levels under PEG treatment in five-week-old *Vitis* spp. cv ‘Venus’. *VvACTIN7* was used as an internal control and compared to expression in 0 h. Values represent the means ± SD from three independent repeats, and different letters indicate significant differences (one-way ANOVA, P < 0.05).

## References

[B1] Ali K. Maltese F. Choi Y. H. Verpoorte R. (2010). Metabolic constituents of grapevine and grape-derived products. Phytochem. Rev. 9, 357–378. doi: 10.1007/s11101-009-9158-0 20835385 PMC2928446

[B2] Apel K. Hirt H. (2004). Reactive oxygen species: metabolism, oxidative stress, and signal transduction. Annu. Rev. Plant Biol. 55, 373–399. doi: 10.1146/annurev.arplant.55.031903.141701 15377225

[B3] Bi C. Yu Y. Dong C. Yang Y. Zhai Y. Du F.. (2021). The bZIP transcription factor *TabZIP15* improves salt stress tolerance in wheat. Plant Biotechnol. J. 19, 209–211. doi: 10.1111/pbi.13453 32702168 PMC7868967

[B4] Briz-Cid N. Pose-Juan E. Rial-Otero R. Simal-Gándara J. (2016). Proteome changes in garnacha tintorera red grapes during post-harvest drying. LWT-Food Sci. Technol. 69, 608–613. doi: 10.1016/j.lwt.2016.02.026

[B5] Chen J. Mao L. Mi H. Lu W. Ying T. Luo Z. (2016). Involvement of three annexin genes in the ripening of strawberry fruit regulated by phytohormone and calcium signal transduction. Plant Cell Rep. 35, 733–743. doi: 10.1007/s00299-015-1915-5 26724928

[B6] Clark G. B. Morgan R. O. Fernandez M. P. Roux S. J. (2012). Evolutionary adaptation of plant annexins has diversified their molecular structures, interactions and functional roles. New Phytol. 196, 695–712. doi: 10.1111/j.1469-8137.2012.04308.x 22994944

[B7] Corrêa L. G. Riaño-Pachón D. M. Schrago C. G. Dos Santos R. V. Mueller-Roeber B. Vincentz M. (2008). The role of bZIP transcription factors in green plant evolution: adaptive features emerging from four founder genes. PloS One 3, e2944. doi: 10.1371/journal.pone.0002944 18698409 PMC2492810

[B8] Cruz De Carvalho M. H. (2008). Drought stress and reactive oxygen species: Production, scavenging and signaling. Plant Signal Behav. 3, 156–165. doi: 10.4161/psb.3.3.5536 19513210 PMC2634109

[B9] Demidchik V. Shabala S. Isayenkov S. Cuin T. A. Pottosin I. (2018). Calcium transport across plant membranes: mechanisms and functions. New Phytol. 220, 49–69. doi: 10.1111/nph.15266 29916203

[B10] Fang L. Su L. Sun X. Li X. Sun M. Karungo S. K.. (2016). Expression of *Vitis amurensis* NAC26 in *Arabidopsis* enhances drought tolerance by modulating jasmonic acid synthesis. J. Exp. Bot. 67, 2829–2845. doi: 10.1093/jxb/erw122 27162276 PMC4861026

[B11] Gao S. Song T. Han J. He M. Zhang Q. Zhu Y.. (2020). A calcium-dependent lipid binding protein, *OsANN10*, is a negative regulator of osmotic stress tolerance in rice. Plant Sci. 293, 110420. doi: 10.1016/j.plantsci.2020.110420 32081268

[B12] He F. Gao C. Guo G. Liu J. Gao Y. Pan R.. (2019). Maize annexin genes *ZmANN33* and *ZmANN35* encode proteins that function in cell membrane recovery during seed germination. J. Exp. Bot. 70, 1183–1195. doi: 10.1093/jxb/ery452 30649398 PMC6382337

[B13] He X. Liao L. Xie S. Yao M. Xie P. Liu W.. (2020). Comprehensive analyses of the annexin (ANN) gene family in *Brassica rapa*, *Brassica oleracea* and *Brassica napus* reveals their roles in stress response. Sci. Rep. 10, 4295. doi: 10.1038/s41598-020-59953-w 32152363 PMC7062692

[B14] Hou L. Fan X. Hao J. Liu G. Zhang Z. Liu X. (2020). Negative regulation by transcription factor *VvWRKY13* in drought stress of *Vitis vinifera l* . Plant Physiol. Bioch. 148, 114–121. doi: 10.1016/j.plaphy.2020.01.008 31954281

[B15] Hussain H. A. Men S. Hussain S. Chen Y. Ali S. Zhang S.. (2019). Interactive effects of drought and heat stresses on morpho-physiological attributes, yield, nutrient uptake and oxidative status in maize hybrids. Sci. Rep. 9, 3890. doi: 10.1038/s41598-019-40362-7 30846745 PMC6405865

[B16] Jami S. K. Clark G. B. Ayele B. T. Ashe P. Kirti P. B. (2012). Genome-wide comparative analysis of annexin superfamily in plants. PloS One 7, e47801. doi: 10.1371/journal.pone.0047801 23133603 PMC3487801

[B17] Kooyers N. J. (2015). The evolution of drought escape and avoidance in natural herbaceous populations. Plant Sci. 234, 155–162. doi: 10.1016/j.plantsci.2015.02.012 25804818

[B18] Laohavisit A. Davies J. M. (2011). Annexins. New Phytol. 189, 40–53. doi: 10.1111/j.1469-8137.2010.03533.x 21083562

[B19] Laohavisit A. Richards S. L. Shabala L. Chen C. Colaço R. D. Swarbreck S. M.. (2013). Salinity-induced calcium signaling and root adaptation in *Arabidopsis* require the calcium regulatory protein annexin1. Plant Physiol. 163, 253–262. doi: 10.1104/pp.113.217810 23886625 PMC3762646

[B20] Lee N. Wetzstein H. Y. (1988). Protoplast isolation and callus production from leaves of tissue-cultured *Vitis* spp. Plant Cell Rep. 7, 531–534. doi: 10.1007/bf00272749 24240409

[B21] Li Z. Fu D. Wang X. Zeng R. Zhang X. Tian J.. (2022). The transcription factor bZIP68 negatively regulates cold tolerance in maize. Plant Cell. 34, 2833–2851. doi: 10.1093/plcell/koac137 35543494 PMC9338793

[B22] Li D. Fu F. Zhang H. Song F. (2015). Genome-wide systematic characterization of the bZIP transcriptional factor family in tomato (*Solanum lycopersicum l.*). BMC Genom. 16, 771. doi: 10.1186/s12864-015-1990-6 PMC4603586 26459863

[B23] Li Z. Tang J. Srivastava R. Bassham D. C. Howell S. H. (2020). The transcription factor bZIP60 links the unfolded protein response to the heat stress response in maize. Plant Cell. 32, 3559–3575. doi: 10.1105/tpc.20.00260 32843434 PMC7610289

[B24] Li G. Xu W. Jing P. Hou X. Fan X. (2021). Overexpression of *VyDOF8*, a Chinese wild grapevine transcription factor gene, enhances drought tolerance in transgenic tobacco. Environ. Exp. Bot. 190, 104592. doi: 10.1016/j.envexpbot.2021.104592

[B25] Liao C. Zheng Y. Guo Y. (2017). MYB30 transcription factor regulates oxidative and heat stress responses through ANNEXIN-mediated cytosolic calcium signaling in *Arabidopsis* . New Phytol. 216, 163–177. doi: 10.1111/nph.14679 28726305

[B26] Liu J. Chen N. Chen F. Cai B. Dal Santo S. Tornielli G. B.. (2014). Genome-wide analysis and expression profile of the bZIP transcription factor gene family in grapevine (*Vitis vinifera*). BMC Genom. 15, 281. doi: 10.1186/1471-2164-15-281 PMC4023599 24725365

[B27] Liu J. Chu J. Ma C. Jiang Y. Ma Y. Xiong J.. (2019). Overexpression of an ABA-dependent grapevine bZIP transcription factor, *VvABF2*, enhances osmotic stress in *Arabidopsis* . Plant Cell Rep. 38, 587–596. doi: 10.1007/s00299-019-02389-y 30712103

[B28] Liu Q. Ding Y. Shi Y. Ma L. Wang Y. Song C.. (2021b). The calcium transporter ANNEXIN1 mediates cold-induced calcium signaling and freezing tolerance in plants. EMBO J. 40, e104559. doi: 10.15252/embj.2020104559 33372703 PMC7809786

[B29] Liu C. Kang H. Wang Y. Yao Y. Gao Z. Du Y. (2021a). Melatonin relieves ozone stress in grape leaves by inhibiting ethylene biosynthesis. Front. Plant Sci. 12. doi: 10.3389/fpls.2021.702874 PMC8355546 34394155

[B30] Lovisolo C. Perrone I. Carra A. Ferrandino A. Flexas J. Medrano H.. (2010). Drought-induced changes in development and function of grapevine (*Vitis* spp.) organs and in their hydraulic and non-hydraulic interactions at the whole-plant level: A physiological and molecular update. Funct. Plant Biol. 37, 98–116. doi: 10.1071/FP09191

[B31] Mahmood T. Khalid S. Abdullah M. Ahmed Z. Shah M. K. N. Ghafoor A.. (2019). Insights into drought stress signaling in plants and the molecular genetic basis of cotton drought tolerance. Cells. 9, 105. doi: 10.3390/cells9010105 31906215 PMC7016789

[B32] Miller G. Suzuki N. Ciftci-Yilmaz S. Mittler R. (2010). Reactive oxygen species homeostasis and signalling during drought and salinity stresses. Plant Cell Environ. 33, 453–467. doi: 10.1111/j.1365-3040.2009.02041.x 19712065

[B33] Mittler R. Vanderauwera S. Gollery M. Van Breusegem F. (2004). Reactive oxygen gene network of plants. Trends Plant Sci. 9, 490–498. doi: 10.1016/j.tplants.2004.08.009 15465684

[B34] Mortimer J. C. Laohavisit A. Macpherson N. Webb A. Brownlee C. Battey N. H.. (2008). Annexins: multifunctional components of growth and adaptation. J. Exp. Bot. 59, 533–544. doi: 10.1093/jxb/erm344 18267940

[B35] Nicolas P. Lecourieux D. Kappel C. Cluzet S. Cramer G. Delrot S.. (2014). The basic leucine zipper transcription factor ABSCISIC ACID RESPONSE ELEMENT-BINDING FACTOR2 is an important transcriptional regulator of abscisic acid-dependent grape berry ripening processes. Plant Physiol. 164, 365–383. doi: 10.1104/pp.113.231977 24276949 PMC3875815

[B36] Nijhawan A. Jain M. Tyagi A. K. Khurana J. P. (2008). Genomic survey and gene expression analysis of the basic leucine zipper transcription factor family in rice. Plant Physiol. 146, 333–350. doi: 10.1104/pp.107.112821 18065552 PMC2245831

[B37] Pan R. Buitrago S. Feng Z. Abou-Elwafa S. F. Xu L. Li C.. (2022). *HvbZIP21*, a novel transcription factor from wild barley confers drought tolerance by modulating ROS scavenging. Front. Plant Sci. 13. doi: 10.3389/fpls.2022.878459 PMC9074790 35528943

[B38] Qiao B. Zhang Q. Liu D. Wang H. Yin J. Wang R.. (2015). A calcium-binding protein, rice annexin *OsANN1*, enhances heat stress tolerance by modulating the production of H_2_O_2_ . J. Exp. Bot. 66, 5853–5866. doi: 10.1093/jxb/erv294 26085678

[B39] Rescher U. Gerke V. (2004). Annexins–unique membrane binding proteins with diverse functions. J. Cell Sci. 117, 2631–2639. doi: 10.1242/jcs.01245 15169834

[B40] Romero P. Dodd I. C. Martinez-Cutillas A. (2012). Contrasting physiological effects of partial root zone drying in field-grown grapevine (*Vitis vinifera l.* cv. monastrell) according to total soil water availability. J. Exp. Bot. 63, 4071–4083. doi: 10.1093/jxb/ers088 22451721 PMC3398444

[B41] Saad R. B. Ben Romdhane W. Ben Hsouna A. Mihoubi W. Harbaoui M. Brini F. (2020). Insights into plant annexins function in abiotic and biotic stress tolerance. Plant Signal Behav. 15, 1699264. doi: 10.1080/15592324.2019.1699264 31822147 PMC7012142

[B42] Shen F. Ying J. Xu L. Sun X. Wang J. Wang Y.. (2021). Characterization of annexin gene family and functional analysis of *RsANN1a* involved in heat tolerance in radish (*Raphanus sativus l.*). Physiol. Mol. Biol. Plants 27, 2027–2041. doi: 10.1007/s12298-021-01056-5 34629776 PMC8484430

[B43] Su L. Fang L. Zhu Z. Zhang L. Sun X. Wang Y.. (2020). The transcription factor *VaNAC17* from grapevine (*Vitis amurensis*) enhances drought tolerance by modulating jasmonic acid biosynthesis in transgenic *Arabidopsis* . Plant Cell Rep. 39, 621–634. doi: 10.1007/s00299-020-02519-x 32107612

[B44] Tong T. Li Q. Jiang W. Chen G. Xue D. Deng F.. (2021). Molecular evolution of calcium signaling and transport in plant adaptation to abiotic stress. Int. J. Mol. Sci. 22, 22. doi: 10.3390/ijms222212308 PMC8618852 34830190

[B45] Wang H. Wang W. Zhan J. Huang W. Xu H. J. S. H. (2015). An efficient PEG-mediated transient gene expression system in grape protoplasts and its application in subcellular localization studies of flavonoids biosynthesis enzymes. Sci. Hortic. 191, 82–89. doi: 10.1016/j.scienta.2015.04.039

[B46] Waseem M. Rong X. Li Z. (2019). Dissecting the role of a basic helix-loop-helix transcription factor, *SlbHLH22*, under salt and drought stresses in transgenic *Solanum lycopersicum l* . Front. Plant Sci. 10. doi: 10.3389/fpls.2019.00734 PMC6558761 31231412

[B47] Wei K. Chen J. Wang Y. Chen Y. Chen S. Lin Y.. (2012). Genome-wide analysis of bZIP-encoding genes in maize. DNA Res. 19, 463–476. doi: 10.1093/dnares/dss026 23103471 PMC3514857

[B48] Xi Y. Liu J. Dong C. Cheng Z. M. (2017). The CBL and CIPK gene family in grapevine (*Vitis vinifera*): Genome-wide analysis and expression profiles in response to various abiotic stresses. Front. Plant Sci. 8. doi: 10.3389/fpls.2017.00978 PMC5465270 28649259

[B49] Xiang Y. Tang N. Du H. Ye H. Xiong L. (2008). Characterization of *OsbZIP23* as a key player of the basic leucine zipper transcription factor family for conferring abscisic acid sensitivity and salinity and drought tolerance in rice. Plant Physiol. 148, 1938–1952. doi: 10.1104/pp.108.128199 18931143 PMC2593664

[B50] Xiong H. Yu J. Miao J. Li J. Zhang H. Wang X.. (2018). Natural variation in *OsLG3* increases drought tolerance in rice by inducing ROS scavenging. Plant Physiol. 178, 451–467. doi: 10.1104/pp.17.01492 30068540 PMC6130013

[B51] Xu Z. Y. Kim S. Y. Hyeon Do Y. Kim D. H. Dong T. Park Y.. (2013). The *Arabidopsis* NAC transcription factor ANAC096 cooperates with bZIP-type transcription factors in dehydration and osmotic stress responses. Plant Cell. 25, 4708–4724. doi: 10.1105/tpc.113.119099 24285786 PMC3875745

[B52] Xu W. Shen W. Ma J. Ya R. Zheng Q. Wu N.. (2020). Role of an amur grape CBL-interacting protein kinase VaCIPK02 in drought tolerance by modulating ABA signaling and ROS production. Environ. Exp. Bot. 172, 103999. doi: 10.1016/j.envexpbot.2020.103999

[B53] Yamaguchi N. Winter C. M. Wu M. F. Kwon C. S. William D. A. Wagner D. (2014). PROTOCOLS: Chromatin immunoprecipitation from *Arabidopsis* tissues. Arabidopsis Book. 12, e0170. doi: 10.1199/tab.0170 24653666 PMC3952383

[B54] Yang S. Xu K. Chen S. Li T. Xia H. Chen L.. (2019). A stress-responsive bZIP transcription factor *OsbZIP62* improves drought and oxidative tolerance in rice. BMC Plant Biol. 19, 260. doi: 10.1186/s12870-019-1872-1 31208338 PMC6580479

[B55] You J. Chan Z. (2015). ROS regulation during abiotic stress responses in crop plants. Front. Plant Sci. 6. doi: 10.3389/fpls.2015.01092 PMC4672674 26697045

[B56] Zhang Q. Song T. Guan C. Gao Y. Ma J. Gu X.. (2021). *OsANN4* modulates ROS production and mediates Ca^(2+)^ influx in response to ABA. BMC Plant Biol. 21, 474. doi: 10.1186/s12870-021-03248-3 34663209 PMC8522085

[B57] Zhang B. Zhang C. Liu C. Jing Y. Wang Y. Jin L.. (2018a). Inner envelope CHLOROPLAST MANGANESE TRANSPORTER 1 supports manganese homeostasis and phototrophic growth in *Arabidopsis* . Mol. Plant 11, 943–954. doi: 10.1016/j.molp.2018.04.007 29734003

[B58] Zhang G. Zhang M. Zhao Z. Ren Y. Li Q. Wang W. (2017). Wheat TaPUB1 modulates plant drought stress resistance by improving antioxidant capability. Sci. Rep. 7, 7549. doi: 10.1038/s41598-017-08181-w 28790447 PMC5548723

[B59] Zhang Y. Zhao H. Zhou S. He Y. Luo Q. Zhang F.. (2018b). Expression of *TaGF14b*, a 14-3-3 adaptor protein gene from wheat, enhances drought and salt tolerance in transgenic tobacco. Planta 248, 117–137. doi: 10.1007/s00425-018-2887-9 29616395

[B60] Zhao W. Wang X. Zhang Q. Zheng Q. Yao H. Gu X.. (2022). H3K36 demethylase *JMJ710* negatively regulates drought tolerance by suppressing *MYB48-1* expression in rice. Plant Physiol. 189, 1050–1064. doi: 10.1093/plphys/kiac095 35253881 PMC9157158

[B61] Zhou Y. B. Liu C. Tang D. Y. Yan L. Wang D. Yang Y. Z.. (2018). The receptor-like cytoplasmic kinase STRK1 phosphorylates and activates CatC, thereby regulating H_2_O_2_ homeostasis and improving salt tolerance in rice. Plant Cell. 30, 1100–1118. doi: 10.1105/tpc.17.01000 29581216 PMC6002193

[B62] Zhu J. K. (2016). Abiotic stress signaling and responses in plants. Cell. 167, 313–324. doi: 10.1016/j.cell.2016.08.029 27716505 PMC5104190

