# Supplementary material for: Grapevine bZIP transcription factor bZIP45 regulates VvANN1 and confers drought tolerance in Arabidopsis
Source: Front Plant Sci. 2023 Feb 9;14:1128002. doi: 10.3389/fpls.2023.1128002 (PMC9947540; doi:10.3389/fpls.2023.1128002)
Supplement: Supplementary file 1 [file Table_1.doc]

**Supplementary Table 1**

**List of primers used in this study**

| **A. Primers used in vector construction** | | |
| --- | --- | --- |
| vectors | Forward primer (5’-3’) | Reverse primer (5’-3’) |
| Ubi::*VvANN1*-HA | TTCTGCAGCGGGATCCATGGCGACTCTTTCCGTGACCG | CGTAACGCGTGGATCCTTAGCATCTCCGTGTCCTATCAG |
| 35S::*VvANN1*-GFP | TCTAGAATGGCGACTCTTTCCGTGACCG | GGATCCAGCATCTCCGTGTCCTATCAG |
| *VvANN1*-His | GGATCCATGGCGACTCTTTCCGTGACCG | GAGCTCTCAAGCATCTCCGTGTCCTATCAG |
| *VvANN1Pro*::GUS | CTGCAGAGGGTCTAACTCCCCACTAAGC | GGATCCCCTTTTCACTGTTTCCTGG |
| pAbAi-*VvANN1Pro* | AAGCTTGACAGAGTCCTTATCGCACC | GTCGACCCTTTTCACTGTTTCCTGG |
| pGreenII0800-LUC-*VvANN1Pro* | AAGCTTGACAGAGTCCTTATCGCACC | GGATCCCCTTTTCACTGTTTCCTGG |
| Ubi::*VvbZIP45*-HA | TTCTGCAGCGGGATCCATGGGGAGTAATTTGAACTTCA | CGTAACGCGTGGATCCAACCAGGGGCCAGTCAGTGT |
| pGreenII62-SK-*VvbZIP45* | GAATTCATGGGGAGTAATTTGAACTTCA | GGATCCTCACCAGGGGCCAGTCAGTGT |
| AD-*VvbZIP45* | GAATTCATGGGGAGTAATTTGAACTTCA | GGATCCTCACCAGGGGCCAGTCAGTGT |
| 35S::*VvbZIP45*-GFP | TCTAGAATGGGGAGTAATTTGAACTTCA | GGATCCCCAGGGGCCAGTCAGTGTGC |
|  | | |
| **B. Primers used in ChIP-qPCR analysis** | | |
| Primer name | Forward primer (5’-3’) | Reverse primer (5’-3’) |
| *VvANN1* promoter-1 | CATCACCAATTTTTCTGCCCACTTTA | GGAGCCACACTTCCAGCGTCAC |
| *VvANN1* promoter-2 | ACTTCTCATGTCCATCCTTCCCTAA | TGAAAGATGGGTTTGAAAAGGTGAC |
| *VvANN1* promoter-3 | CTTCGACAGAGTCCTTATCGCACC | TTTCTCGCCGCCACGTCACA |
| *VvANN1* promoter-4 | GAAATTCAGAAGGATCATCCCGTAT | TGTTTCCTGGACAAGGTAAGGTTTT |
|  | | |
| **C. Primers used in real time PCR analysis** | | |
| Gene | Forward primer (5’-3’) | Reverse primer (5’-3’) |
| *VvANN1* | AAGTGCTTGACCCGCCCAGAGA | CCAGAGTGACGCTGTTCCTTTTA |
| *VvbZIP45* | CGCTGACCTTTGACGAGTT | CTTCAGCACTCCAAATGTTCTT3 |
| *VvACTIN7* | GTGCCTGCCATGTATGTTGCC | GGTCACGTCCAGCAAGGTCAAG |
| *AtACTIN2* | TCTCTTCCTCATGCCATCCTCC | CTCTTACAATTTCCCGCTCTGC |
