# Supplementary figures and images for: Grapevine bZIP transcription factor bZIP45 regulates VvANN1 and confers drought tolerance in Arabidopsis
Source: Front Plant Sci. 2023 Feb 9;14:1128002. doi: 10.3389/fpls.2023.1128002 (PMC9947540; doi:10.3389/fpls.2023.1128002)

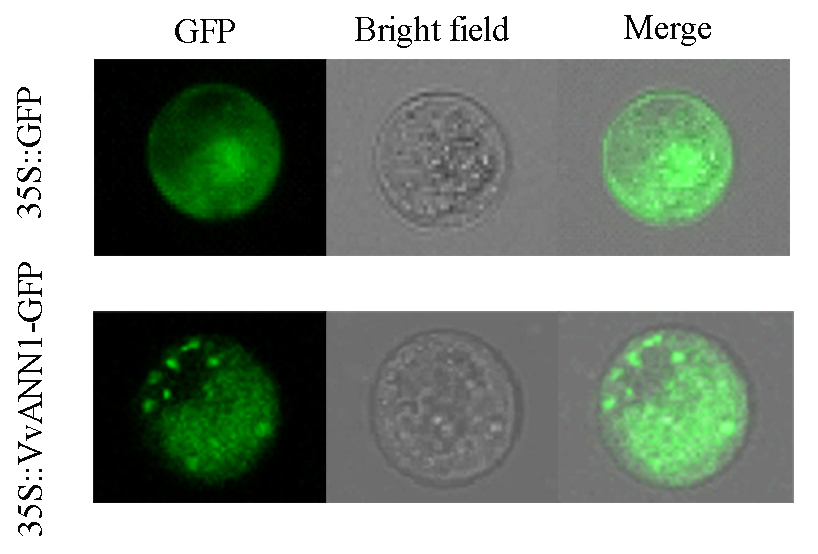

Supplement: Supplementary Figure 1 — Subcellular localization of VvANN1-GFP. Subcellular localization of VvANN1-GFP in grape protoplasts. Protoplasts transformed with 35S::GFP were used as control. Scale bars=20 μm. [file Image_1.jpeg]

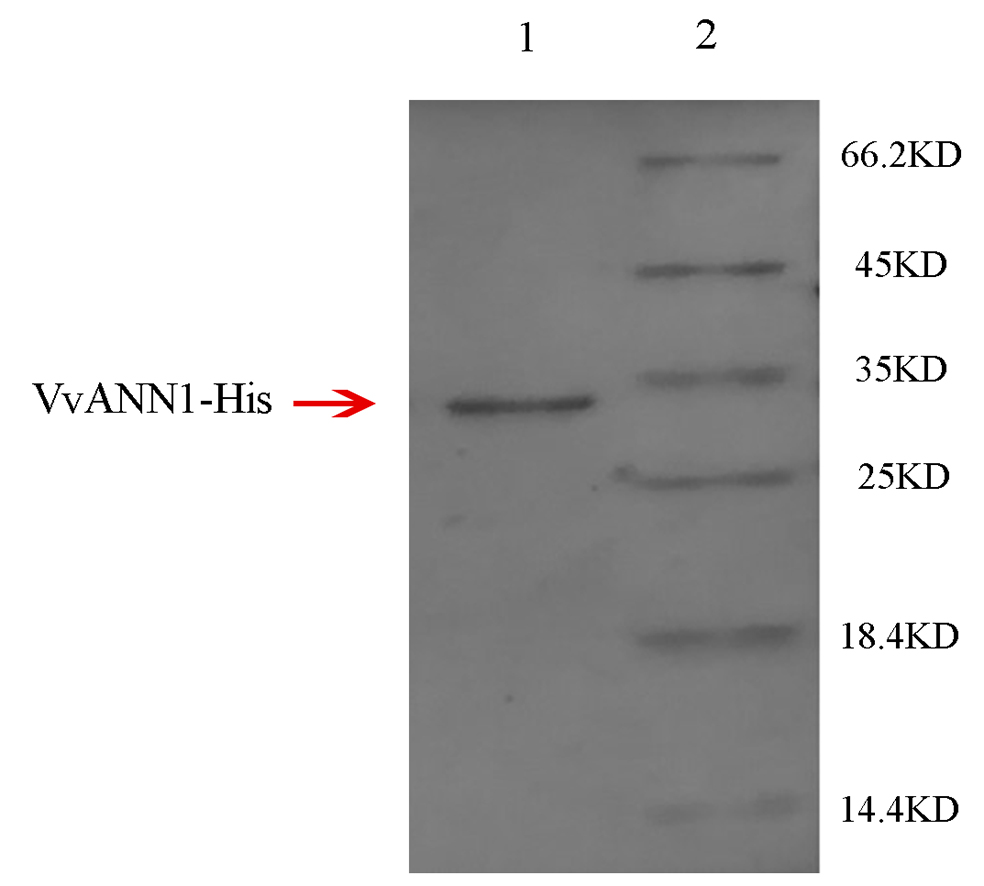

Supplement: Supplementary Figure 2 — Affinity purification of VvANN1-His protein. Purification of VvANN1-His recombinant protein was performed with 0.2 mL elution buffer containing 500 mM imidazole (lane 1), lane 2 shows the molecular weight marker. [file Image_2.jpeg]

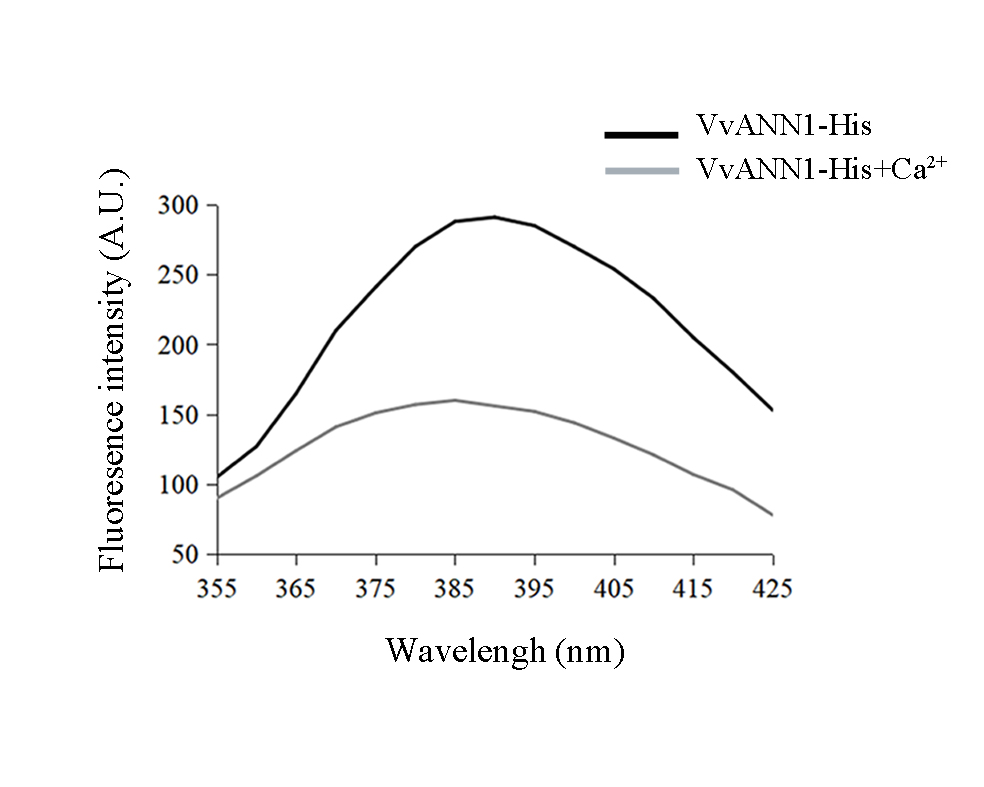

Supplement: Supplementary Figure 3 — The Ca2+-binding capacity of VvANN1-His. Fluorescence intensity of VvANN1-His without (black curve) or with (grey curve) the addition of 2 mM CaCl2. [file Image_3.jpeg]

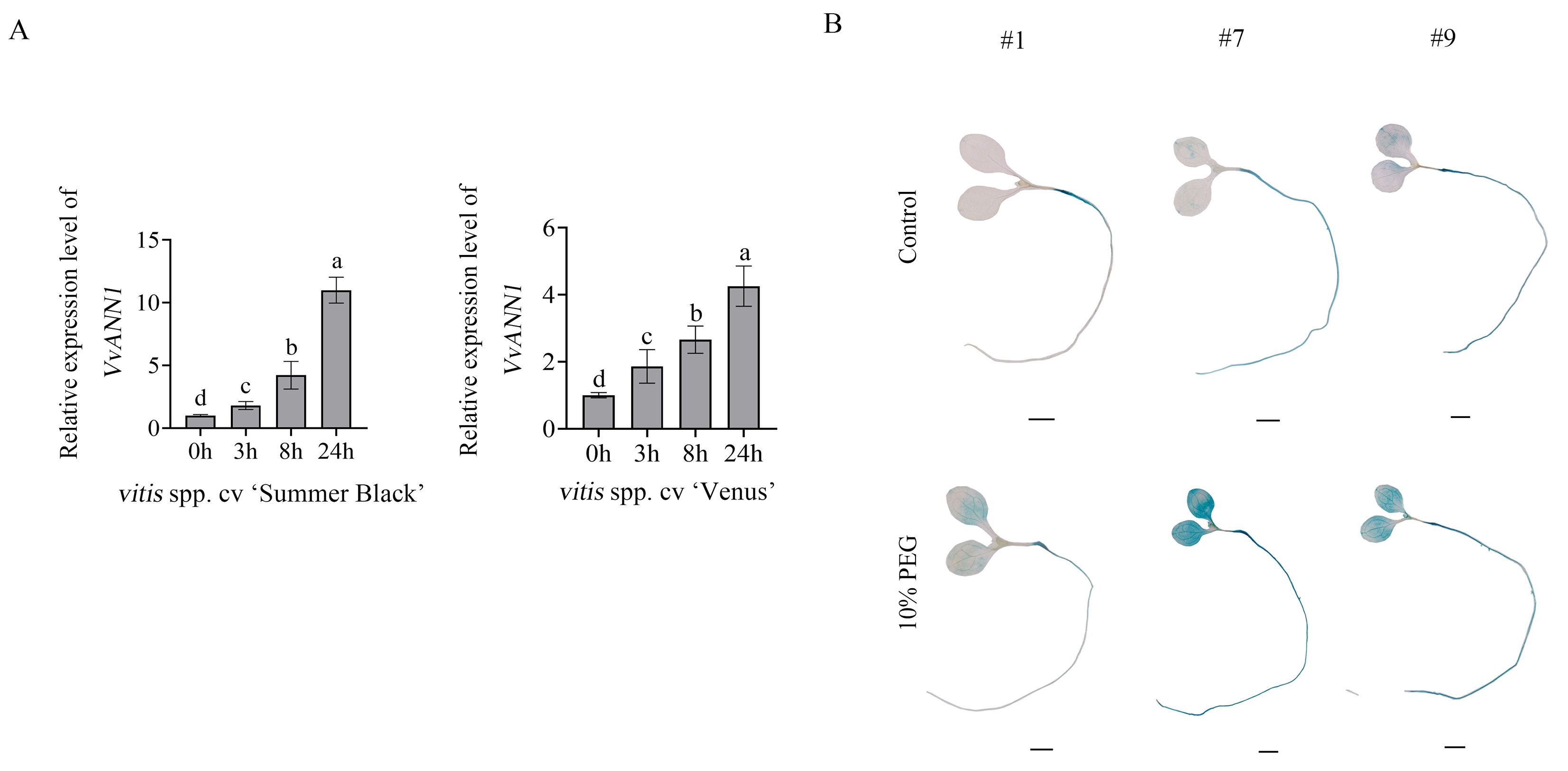

Supplement: Supplementary Figure 4 — Expression patterns of VvANN1 under PEG treatment. (A) RT-qPCR analysis of VvANN1 expression in the five-week-old Vitis spp. cv ‘Summer Black’ and ‘Venus’ plantlets after 10% PEG6000 treatment. VvACTIN7 was used as an internal control and compared to expression in 0 h. Values represent the means ± SD from three independent repeats, and different letters indicate significant differences (one-way ANOVA, P<0.05). (B) GUS staining of six-day-old VvANN1Pro::GUS transgenic Arabidopsis seedlings under normal and PEG treatment. Scale bars=1 mm. [file Image_4.jpeg]

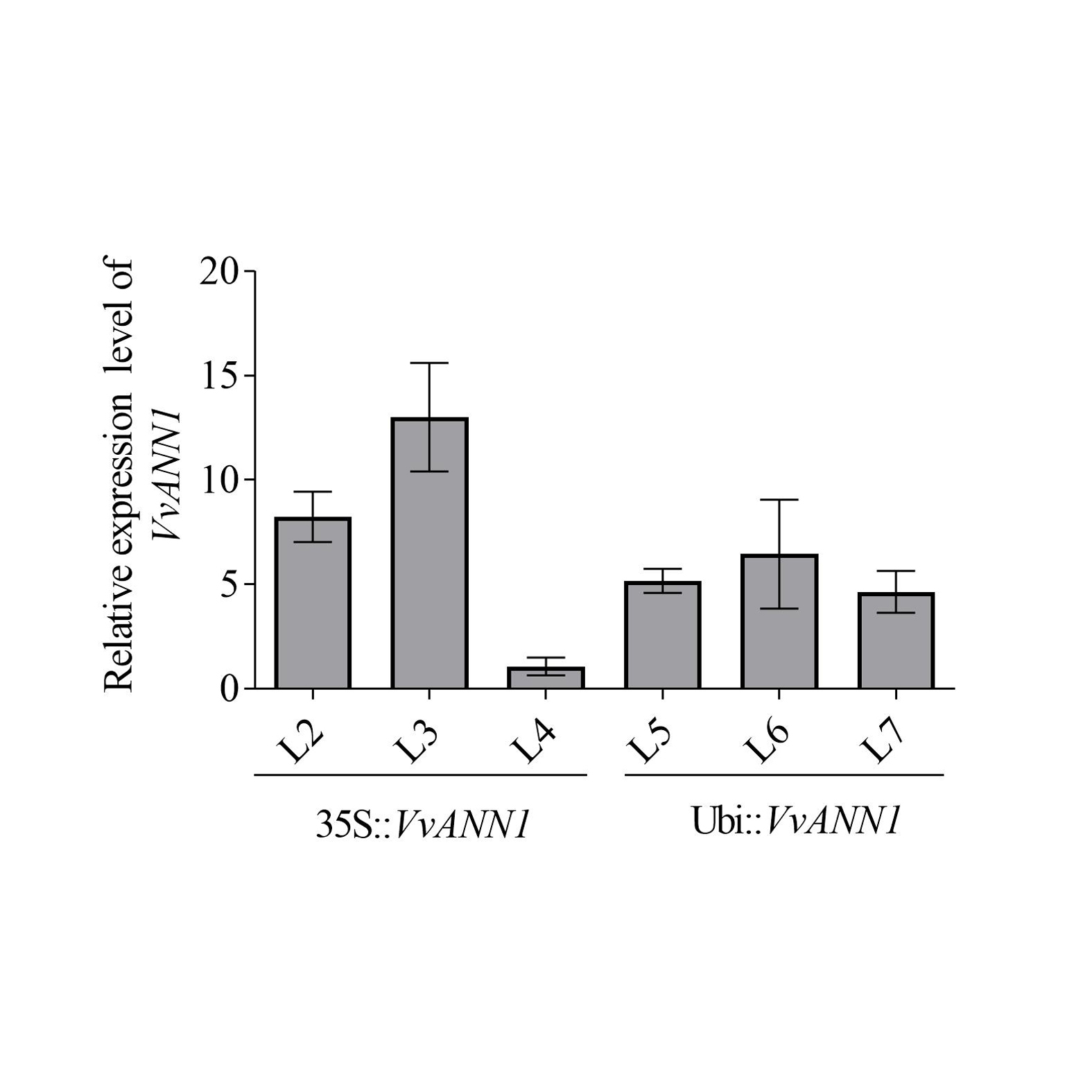

Supplement: Supplementary Figure 5 — RT-qPCR analysis of VvANN1 expression in different VvANN1 transgenic Arabidopsis lines. AtACTIN2 was used as an internal control. Values represent the means ± SD from three independent repeats. [file Image_5.jpeg]

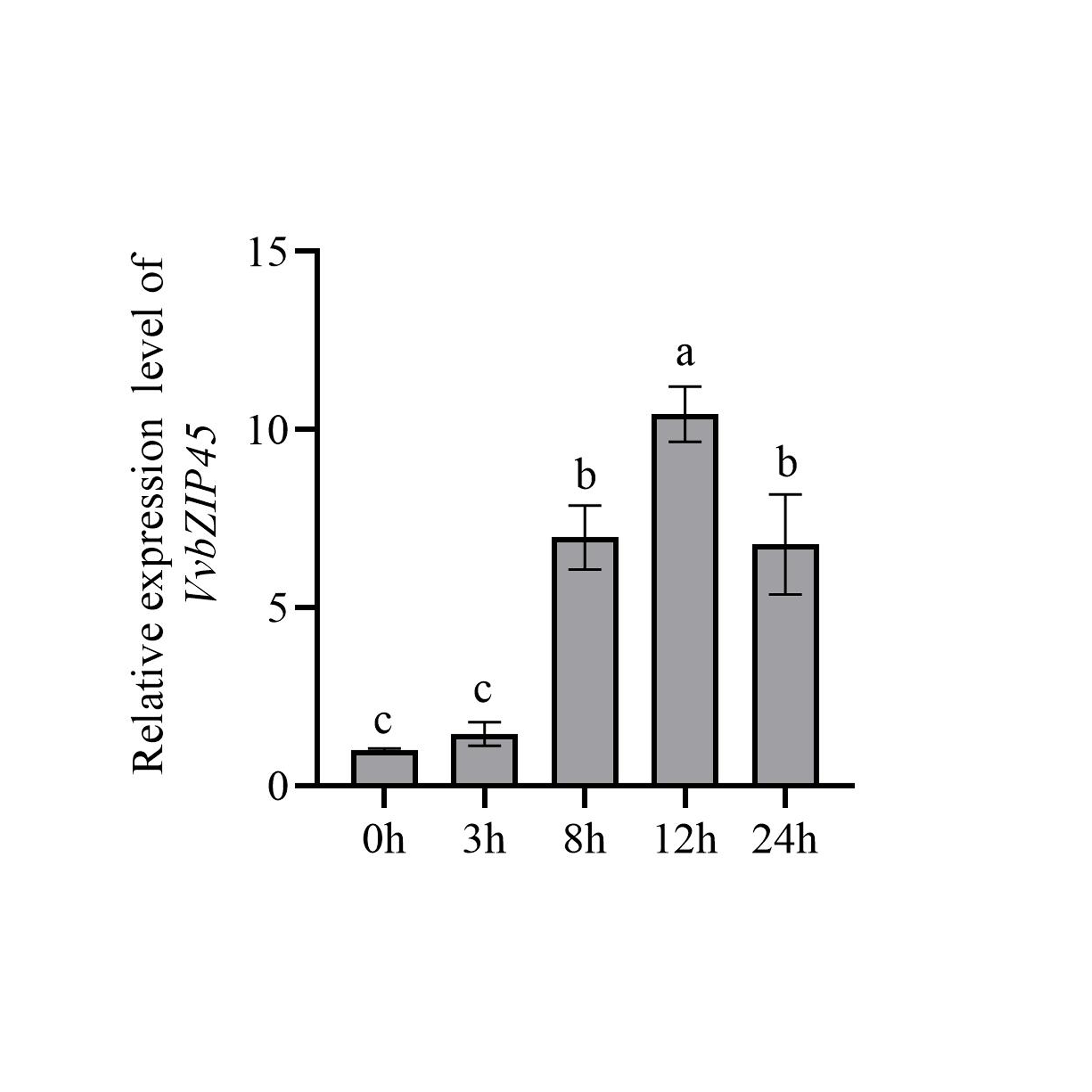

Supplement: Supplementary Figure 6 — VvbZIP45 transcript expression levels under PEG treatment in five-week-old Vitis spp. cv ‘Venus’. VvACTIN7 was used as an internal control and compared to expression in 0 h. Values represent the means ± SD from three independent repeats, and different letters indicate significant differences (one-way ANOVA, P < 0.05). [file Image_6.jpeg]
